# Biodegradation of Water-Soluble Polymers by Wastewater Microorganisms: Challenging Laboratory Testing Protocols

**DOI:** 10.1021/acs.est.4c05808

**Published:** 2024-08-12

**Authors:** Aaron Kintzi, Soumya Daturpalli, Glauco Battagliarin, Michael Zumstein

**Affiliations:** † Division of Environmental Geosciences, Centre for Microbiology and Environmental Systems Science, University of Vienna, Josef-Holaubek-Platz 2, Vienna 1090, Austria; ‡ Doctoral School in Microbiology and Environmental Science, University of Vienna, Vienna 1090, Austria; § BASF SE, Ludwigshafen am Rhein 67056, Germany

**Keywords:** Water-soluble polymers, biodegradation testing, biological wastewater treatment, environmentally benign-by-design

## Abstract

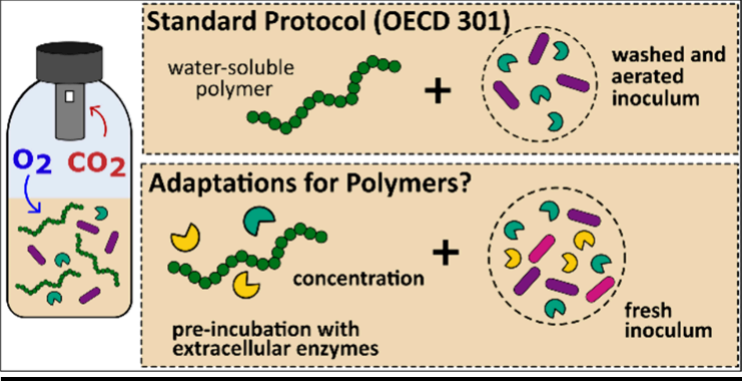

For water-soluble polymers (WSPs) that enter environmental systems at their end-of-life, biodegradability is a key functionality. For the development and regulation of biodegradable WSPs, testing methods that are both scientifically validated and economically practicable are needed. Here, we used respirometric laboratory tests to study the biodegradation of poly(amino acids), poly(ethylene glycol), and poly(vinyl alcohol), together with appropriate low-molecular-weight reference substrates. We varied key protocol steps of commonly used testing methods, which were originally established for small molecules and tested for effects on WSP biodegradation. We found that avoiding aeration of the wastewater inoculate prior to WSP addition, incubating WSP with filter-sterilized wastewater prior to biodegradation testing, and lowering the WSP concentration can increase biodegradation rates of WSPs. Combining the above-mentioned protocol variations substantially affected the results of the biodegradation testing for the two poly(amino acids) tested herein (i.e., poly(lysine) and poly(aspartic acid)). Our findings were consistent between microbial inocula derived from two municipal wastewater treatment plants. Our study presents promising biodegradation dynamics for poly(amino acids) and highlights the importance, strengths, and limitations of respirometric laboratory methods for WSP biodegradation testing.

## Abstract 2

We assessed the biodegradation of promising water-soluble polymers and challenged biodegradation testing protocols. Our study provides process insights that are important for the development and testing of biodegradable polymers.

## Introduction

Water-soluble polymers (WSPs) play important roles in many areas of modern societies–including, home and personal care, water and wastewater treatment, and agriculture.^1,2^ WSPs in home care applications act as cleaning agents that remove dirt from dishes and fabrics, or as builders that inhibit encrustation and remove calcium and magnesium from hard water.^1^ The market demand for WSPs is expected to further increase.^1^ After many applications, WSPs are released into wastewater systems.^3−6^ To prevent the input of WSPs into natural environments, biodegradability during wastewater treatment is a key functionality of WSPs used in such applications–particularly because the recovery of WSPs from complex matrices is not feasible.^1,2,7−9^

Biodegradation under aerobic conditions refers to the transformation of organic compounds by living organisms into CO_2_, H_2_O, and biomass.^7,10,11^ The biodegradation of polymers is considered a two-step process. In the first step, which can be catalyzed by extracellular enzymes, the polymer is broken down into intermediates that are small enough for cellular uptake.^11−15^ In the second step, breakdown intermediates are metabolized intracellularly.^10,11^ Importantly, biodegradation depends on both the substance of interest and the respective environment.^10,12,16^ Therefore, it is essential that biodegradation tests are conducted, and their results discussed, in the context of the relevant environmental system.

Experimental testing of biodegradation provides key insights into process steps and factors that govern biodegradation and is needed for regulatory assessments.^17,18^ Obtained insights are furthermore essential for the development of new biodegradable chemicals such as WSPs.^10^ In addition to scientific validity, practicality is an important criterion for testing approaches. Laboratory tests that enable the quantification of biodegradation end points, allow a reasonable experimental throughput, and do not depend on highly specialized laboratory infrastructure or expensive chemicals and materials, are key components of tiered biodegradation testing schemes.^19,20^ Therefore, such laboratory tests are often conducted according to the OECD 301 testing guidelines.^21^ In these controlled tests, which aim at comparative assessments of biodegradation, but do not provide biodegradation rates for real scenarios (e.g., due to the needed high substrate concentrations and low microbial cell concentrations),^22−24^ the substance of interest is incubated with a microbial inoculum derived from the environment of interest in an otherwise carbon-free, pH-buffered mineral medium.^21,25^ During incubation, the amount of CO_2_ produced (direct measurement) and/or the amount of molecular oxygen (O_2_) consumed (indirect measurement) are quantified in real time. The extent of biodegradation is inferred from these measurements after subtracting the activity of parallel incubations without the test substance. The experiments are validated by including appropriate reference substances known to be biodegraded.^20^ Besides demonstrating activity of the microbial inocula, reference substrates provide insights into possible differences in carbon use efficiencies across substrates.^12^

Water-soluble polymers, for which promising rates and extents of biodegradation were reported using respirometric laboratory methods based on wastewater microbiomes, include poly(ethylene glycol) (PEG),^25−29^ poly(vinyl alcohol) (PVA),^25,29−31^ and poly(aspartic acid) (PAsA).^32−35^ While PEG and PVA are already used at large volumes,^2^ poly(amino acids) are considered promising with respect to both function and biodegradability due to the abundance and activity of extracellular peptidases in wastewater systems.^12,36,37^ For example, PAsA has been discussed as a potential replacement of large-volume WSPs such as poly(acrylic acid)^1,7,11,28^ and (cationic) poly(l-lysine) (PLL) is considered promising for numerous applications, including cosmetics.^38−40^

For PEG and PVA, previous respirometric laboratory analyses with microorganisms from WWTP aeration tanks showed that more than 80% of the carbon from these WSPs were converted to CO_2_ within 15 to 30 days of incubation.^25,26,29^ A cross-laboratory comparison, in which microbial inocula from eight different WWTPs was used, showed that biodegradation rates and extents of PEG and PVA were similar across these inocula.^29^ Studies on PAsA, in which the amount of consumed O_2_ during the incubation with wastewater microorganisms was quantified, reported between 60 and 80% biodegradation within 28 days.^34,35^ For ε-poly(l-lysine) (PLL), biodegradation by wastewater microorganisms has not been investigated respirometrically, but its breakdown by certain microbial enzymes has been reported.^41−43^

Importantly, OECD 301 testing guidelines were developed for biodegradability assessments of low-molecular-weight chemicals. One protocol step is the washing and aeration (for up to 7 days) of the wastewater microbial inoculum prior to mixing it with the test substance.^21,25^ This step is conducted to reduce the concentration of organic carbon in the inoculum and thereby reduce background respiration for more sensitive measurements. However, aeration and washing can result in an additional selection pressure on the microbial community and alter the microbial community composition, thereby reducing the representativeness of laboratory testing for real environments.^19,22,23,44,45^ Particularly important for WSPs, aeration and washing might decrease the concentration of extracellular enzymes in the inoculum.^12^

To further develop laboratory tests, it is important to consider the path of a substance entering the receiving environment. Water-soluble polymers used in home and personal care enter WWTPs through the sewer system and interact with the wastewater matrix before reaching the aeration tank. Enzymes in the sewer system (such as extracellular peptidases)^46,47^ might catalyze the breakdown of WSPs,^12^ presumably affecting the rate of WSP biodegradation by microorganisms in the aeration tanks of WWTPs. Because adaptation of microorganisms to substrates is expected to be concentration-dependent,^19^ the concentration of the test substance is another relevant parameter for protocol optimization.

The overall goal of this study was to investigate the biodegradation of WSPs (with a focus on the two promising poly(amino acids) PAsA and PLL) by wastewater microorganisms with respirometric biodegradation tests. Specifically, we aimed at assessing the effect of three selected protocol variations on WSP biodegradation results obtained through such tests. These protocol variations included (i) washing and aeration of the microbial inoculum prior to WSP addition, (ii) incubation of WSPs with filter-sterilized wastewater from WWTP influent (i.e., untreated wastewater) prior to biodegradation experiments, and (iii) incubation with different WSP concentrations. To do so, we incubated WSPs, as well as relevant low-molecular-weight reference substrates, with microbial inocula derived from aeration tanks of two full-scale municipal wastewater treatment plants and quantified the O_2_ consumption and the CO_2_ production during incubation to obtain insights into WSP biodegradation dynamics.

## Materials and Methods

A schematic illustration of the conducted experimental protocols and the corresponding figures can be found in Figure S1.

### Chemicals and Materials

ε-Poly(l-lysine) hydrochloride (ε-PLL, article number: FP14985) was purchased from Biosynth. Poly(ethylene glycol) (PEG, Pluriol E 6000) was provided by BASF. Poly(vinyl alcohol) (PVA, 363073), d-glucose (G8270), aspartic acid (A9256), and l-lysine (L5501) were purchased from Sigma-Aldrich. Synthesis of poly(aspartic acid): homopoly(aspartimide) was synthesized from l-aspartic acid, using 25% (mol/mol) phosphoric acid as a catalyst, at 180 °C and 100 mbar under N_2_. Poly(aspartimide) was hydrolyzed with NaOH to obtain the sodium salt of poly(aspartic acid). Additional parameters of the test substances are provided in Table S1, while information on additional chemicals can be found in Text S1. The method for determining the elemental composition and molecular weight of the polymers is reported in Text S2.

### Biodegradation Testing

Biodegradation tests were conducted with a substrate concentration of 100 mg/L (unless otherwise stated) and an activated sludge concentration of 30 mg total suspended solids (TSS)/L in OECD buffer at pH 7.4 following OECD 301.^21^ We used N-allylthiourea to inhibit nitrification^48^ for practicality reasons, but we acknowledge that an effect of nitrification inhibition on WSP biodegradation cannot be excluded. The exact buffer composition is provided in Text S3. Substrate stock solutions were freshly prepared in OECD buffer at concentrations of 3 or 10 g/L, depending on the substrate solubility, with pH adjusted to 7.4 using hydrochloric acid (HCl) or sodium hydroxide (NaOH). For complete dissolution of PVA, the stock solution (3 g/L) was heated to 80 °C and subjected to vortex mixing. Tests were performed in the dark at 20 ± 1 °C using freshly grab-sampled activated sludge from aeration tanks of two full-scale municipal wastewater treatment plants (WWTPs) located in Austria and Germany (see Table S2 for WWTP details). We included inocula from two independent WWTPs to obtain initial insights into the generalizability of our findings– acknowledging that a larger number is needed in future studies to study variabilities across inocula. After transporting the sludge to the laboratory (<1h), the collected activated sludge was aerated (by constant stirring in open 1 L Schott glass bottles) for 3 days (standard protocol), or 6 days where indicated, at room temperature (standard protocol). For experiments with “fresh” or “washed” sludge, biodegradation experiments started within 3 h after sludge sampling at the WWTP. Prior to the start of an experiment, the TSS content of the sludge was measured gravimetrically (as described previously^49^) and adjusted to 3 g TSS/L. Subsequently, the sludge was spiked to the incubation bottles at a final concentration of 30 mg TSS/L. For sludge washing, 500 mL of fresh sludge was added to a measuring cylinder. Once solids settled to the 150 mL mark, the clear supernatant was discarded, and the volume readjusted to 500 mL with OECD buffer.^25^ Biodegradation experiments were conducted in two laboratories for the two WWTPs and followed slightly different protocols. For WWTP1, the OECD buffer was mixed with sludge and spiked with the substrate right before the experiment. For WWTP2, the OECD buffer was prespiked with the respective substrate in incubation bottles, and the wastewater inoculum was added to the mixture after aeration or washing. The abiotic degradation of the test substances was assessed in sterile-filtered OECD buffer (0.22 μm, PES Millipore Stericup, S2GPU05RE) spiked with test substance but no inoculum.

### Respirometric Analyses and Calculations

Biodegradation was monitored using two respirometric systems: the OxiTop system (Xylem Analytica, Germany) for continuous manometric measurements assessing biological oxygen demand (BOD) in line with the OECD 301F guideline, and the BSBdigi-CO_2_ system (SELUTEC GmbH, Germany), for simultaneous O_2_ and CO_2_ measurements (the latter being in line with the OECD 301 B guideline) (schematic in Figure S2). Both systems operate by trapping CO_2_ from the gas phase in a potassium hydroxide (KOH) solution, with the total CO_2_ binding capacity of the absorbing solution calculated and adjusted accordingly.^18^ The OxiTop system monitors the pressure decrease over time as O_2_ is consumed and CO_2_ is trapped in a 1 M KOH solution. The BSBdigi system tracks CO_2_ production through conductivity changes in the constantly stirred 0.1 M KOH absorbing solution (45 mL). The conductivity electrodes were calibrated by spiking defined amounts of Na_2_CO_3_ solution via a rubber septum into the system′s closed incubation vessel containing 250 mL of a 1 M HCl solution.^50^ The pressure drop caused by the O_2_ consumption activates a connected electrolysis cell (via a manometer), that generates O_2_ to ensure a constant pressure and O_2_ level. The extent of biodegradation for the tested substrates was calculated based on the theoretical oxygen demand (ThOD) and the theoretical carbon dioxide production (ThCO_2_). The ThOD [mg O_2_/mg substrate] was calculated based on the measured or theoretical elemental composition according to the following equation (without nitrification due to inhibitor addition):
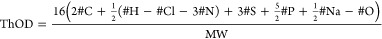
1where # denotes the number of atoms of the respective element in the molecule. MW is the molecular weight of the test chemical. The ThCO_2_ [mg CO_2_/mg substrate] was calculated by the following equation:^51^
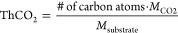
2where *M*_CO2_ and *M*_substrate_ are the molecular mass of CO_2_ and the substrate, respectively. Biodegradation [%] was calculated by correcting O_2_ and CO_2_ measurements for blank values (i.e., incubations without substrate) and dividing by ThOD or ThCO_2_, respectively. The times required to reach 10% or 50% biodegradation were determined from the data of each incubation by extracting the first measurement time point that was equal to or greater than 10% or 50% biodegradation, respectively, using a custom R script. We considered a treatment effect to be substantial and significant when it resulted in a lag-phase increase of ≥1.5 fold (or a lag-phase decrease <0.66 fold) and showed a *p*-value <0.05 in a two-sided *t* test between groups of triplicates. Initial pressure changes in both respirometric systems upon system closure, were occasionally observed, likely due to system equilibration. Any pressure deviations within the first 6 h of an experiment, or within 2 h after an absorber change (i.e., OxiTop system) were considered unrelated to biological activity and set to zero.

### Wastewater Enzyme Extraction and Peptidase Activity Assay

Filter-sterilized wastewater (Figure 2B) was prepared by collecting wastewater directly after the sand trap/rake at the WWTP. At WWTP1, the sample was a daily composite sample consisting of regular automated samples (taken every 4 h) that were stored at 7 °C until pick up. At WWTP2, grab samples were taken. The wastewater was transported to the laboratory within 1 h while being constantly cooled on ice to maintain extracellular enzymes as much as possible. Subsequently, samples were centrifuged (40 mL, 5 min, 2000 *g*) using 50 mL plastic tubes. Following centrifugation, the wastewater was subjected to sterile filtration (0.22 μm, PES Millipore Stericup, S2GPU05RE). Polymers or substrates incubated with filter-sterilized wastewater were prepared by mixing 8.3 mL of substrate (*c* = 3 g/L) with 10 mL of wastewater filtrate in a 50 mL plastic tube. The mixture underwent preincubation at room temperature on a horizontal shaker (150 rpm) for 24 h. In parallel to active extracts, we conducted incubations with autoclaved filtrates to control for abiotic effects such as altered bioavailability of WSPs in response to possible adsorption of WSPs to matrix components in the extract. Filter-sterilized wastewater, rather than whole influent wastewater, was chosen to prevent cells to affect the subsequent biodegradation experiments. For the biodegradation test, the mixture was added to the OECD buffer containing wastewater microbial inoculum.

Filter-sterilized aeration tank samples (Figure 2) were prepared similarly, but enzymes bound to the extracellular polymeric substance (EPS) were additionally targeted by adding 4 g of cation exchange resins (Amberlite HPR1100, 91973) followed by incubating the wastewater suspension on a horizontal shaker (250 rpm) for 30 min prior to centrifugation. Sludge samples undergoing washing with OECD buffer (3.751 mM PO_4_) contained a final buffer concentration of 2.6 mM PO_4_. To ensure comparability, OECD buffer was added to the fresh and aerated sludge in the same concentration. Peptidase activity was determined with the EnzChek Protease Assay kit (Thermo Fisher, E6638) as described previously.^36,49^ 100 μL amount of enzyme extract and 100 μL of freshly prepared working solution, containing the fluorogenic casein substrate, were mixed in a black 96-well microplate (Eppendorf, Microplate 96/U-PP). Fluorescence was quantified with a Tecan Infinite 200 pro plate reader (excitation: 485 nm; emission: 530 nm). We studied the effects of washing and aeration on the sludge microbial community composition by conducting a 16S rRNA gene amplicon sequencing-based analysis of the three sludge samples taken from WWTP1 for biodegradation tests and the one sludge sample taken from WWTP2 for biodegradation tests. Sequencing and data processing were conducted at the Joint Microbiome Facility (Medical University of Vienna and University of Vienna, project ID JMF-23110-02) and are detailed in Text S4.

## Results and Discussion

### Biodegradation of WSPs by Microorganisms from Two WWTPs

In a first step, we assessed the biodegradation of selected WSPs by microorganisms from two WWPTs following the standard OECD 301 F protocol (i.e., using the manometric OxiTop system and including microbial inoculum aeration for 3 days).^21,25^ In parallel to the biodegradation of WSPs, we measured the biodegradation of glucose, lysine, and aspartic acid as reference substrates. While glucose is a common positive control in OECD 301 testing, we included lysine and aspartic acid to mimic breakdown intermediates of PLL and PAsA, respectively.

**Figure 1:**
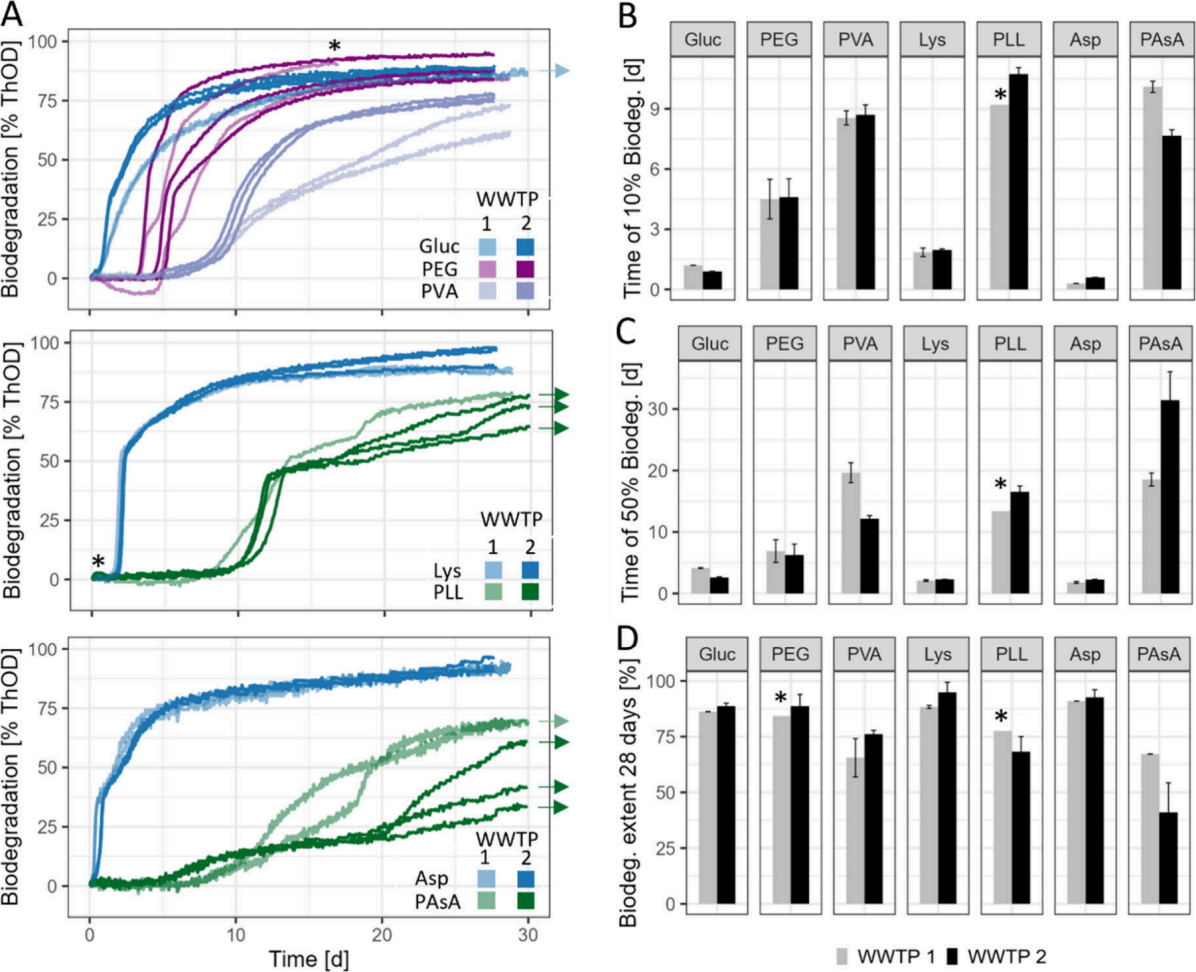
Biodegradation of WSPs by microorganisms derived from two wastewater treatment plants (WWTPs). (A) Biodegradation curves calculated based on theoretical O_2_ demand (ThOD) and measured O_2_ consumption during WSP incubation using the OxiTop system. Arrows indicate extended experiments (complete data in Figure S3). Asterisks (*) indicate stopped incubations due to instrument malfunctioning. (B) and (C) Times to reach 10% and 50% biodegradation, respectively. (D) Biodegradation extents after 28 days of incubation. Error bars represent, where not stated with an asterisk (*), standard deviations of triplicates and ranges of duplicates for WWTP2 and WWTP1, respectively. Gluc: glucose, PEG: poly(ethylene glycol), PVA: poly(vinyl alcohol), Lys: lysine, PLL: ε-poly(l-lysine), Asp: aspartic acid, PAsA: poly(aspartic acid).

The biodegradation of the low-molecular-weight reference substrates was highly reproducible across replicates and inocula. All three substrates reached a biodegradation extent of ∼90% after 28 days (Figure 1). For aspartic acid, we observed a particularly early onset of O_2_ consumption (Figure 1B shows time to reach 10% mineralization, lag phase).^9,21^ In the biodegradation curves of lysine and aspartic acid, we observed a bimodal behavior, with a “kink” at 55 and 40% biodegradation, respectively. This two-stage behavior might reflect different biochemical pathways involved in the biodegradation of these amino acids (e.g., rapid mineralization/oxidation of certain carbon atoms, followed by slower mineralization of carbon atoms initially incorporated into biomolecules).

For all tested WSPs, we detected substantial biodegradation extents, but the variability in biodegradation extents and kinetics across replicates and inocula was larger than for the tested low-molecular-weight substrates. This variability might be linked to the extracellular breakdown of the WSPs. PEG was biodegraded to ∼90% within 28 days, which is consistent with previous studies on PEG biodegradation with a comparable molecular weight.^25,26,29^ Biodegradation curves of PEG were similar between the two WWTPs, with lag phases between 3 and 5 days. For PVA, we observed similar biodegradation kinetics for both inocula during the initial phase of the experiment, with lag phases of ∼9 days. After this time, the biodegradation dynamics diverged between the two WWTPs: 50% biodegradation were reached after 12 days for WWTP2 and after 19 days for WWTP1 (Figure 1C). Similar differences between WWTPs of PVA biodegradation kinetics have been reported before.^25,29^

The two poly(amino acid)s exhibited significantly longer lag phases compared to their respective monomers (Figure 1B). These lag phases can be explained by the time needed for competent organisms to produce (extracellular) enzymes for WSP breakdown or for existing enzymes to break down WSPs into intermediates small enough for cellular uptake. For PLL, the biodegradation dynamic was similar for wastewater microbial inocula from both WWTPs and can be described by three phases: an initial lag phase of ∼10 days, followed by a phase of accelerated biodegradation until ∼45% biodegradation. The third phase is again characterized by slower biodegradation, eventually resulting in a plateau. The final biodegradation extents of PLL at this plateau was ∼80% for both WWTPs (Figure S3). Biodegradation of PAsA differed between the two WWTPs. For WWTP1, biodegradation started after an initial lag phase of ∼7 days, ultimately plateauing at ∼70% biodegradation after 35 days. For WWTP2, biodegradation commenced earlier (lag phase: 5 days), reaching an intermediate plateau at ∼20%. After 20 days, the biodegradation rate increased again, however, with substantial variability across replicates. Extending the experiments beyond 28 days resulted in a plateau at 100% after 60 days (Figure S3).

We note that the applied respirometric method cannot differentiate between biodegradation of the substance of interest and that of organic compounds in the microbial inoculum that are degraded in response to substrate addition (i.e., “priming effect”). Therefore, the extents of biodegradation can be slightly overestimated. For determining exact biodegradation extents, more specialized methods (e.g., based on isotope-labeled polymers)^52−54^ are required.

In addition to the OxiTop system, with which the data discussed above were generated, we employed the BSBdigi-CO_2_ system (hereafter named BSBdigi) enabling the simultaneous monitoring of the consumption of the aqueous O_2_ and the production of CO_2_ during incubation experiments. To compare both end points, we incubated the low-molecular-weight reference substrates (i.e., glucose, lysine, and aspartic acid) and PEG with microbial inocula from WWTP1. Overall, there was good agreement between the biodegradation dynamics derived from O_2_ consumption and CO_2_ production (Figure S4). The extent of biodegradation after a 35-day incubation was slightly lower (i.e., 6–12% points) when assessed using CO_2_ production compared to O_2_ consumption. A small fraction of this deviation (1–2% points) was explained by dissolved CO_2_ in the incubation solution that was thus not trapped in the alkaline solution in which CO_2_ is quantified (Figure S5).^21,55^ The remaining deviation is likely due to processes consuming O_2_ without producing CO_2_ (e.g., partial oxidation of organic chemicals). To assess the comparability of biodegradation results derived with the two systems (i.e., OxiTop and BSBdigi), we conducted parallel experiments for glucose and PEG and observed no substantial differences (Figure S6).

To assess interday variability of the wastewater microbiomes’ potential to biodegrade the selected substrates, we compiled the results of all biodegradation experiments under standard protocol conditions of this study (Figure S7 and S8). For each substrate and for both WWTPs, at least two experiments with inocula sampled at different days were conducted. For both WWTPs, the biodegradation dynamics of glucose, lysine, aspartic acid, PEG, and PVA were very similar across experiments. The largest variability was observed for PLL biodegradation by WWTP1 microorganism (50% biodegradation reached after 16–26 days in one experiment, and after 12–15 days in other experiments) and for PAsA biodegradation by WWTP2 microorganisms (50% biodegradation reached after 18–20 days in one experiment, and after 27–37 days in another). However, biodegradation extents at the end of the tests were consistent between the inoculum sampling days for all substrates and both WWTPs. Abiotic control experiments, conducted over a 20 day period in sterile-filtered OECD buffer without inoculum (Figure S9), showed no mineralization of the tested WSPs. For two of the low-molecular-weight reference substrates (i.e., glucose and aspartic acid) we detected mineralization in these tests, but with substantially longer lag phases compared to the biological tests.

### Effect of Inoculum Washing and Aeration on WSP Biodegradation

**Figure 2:**
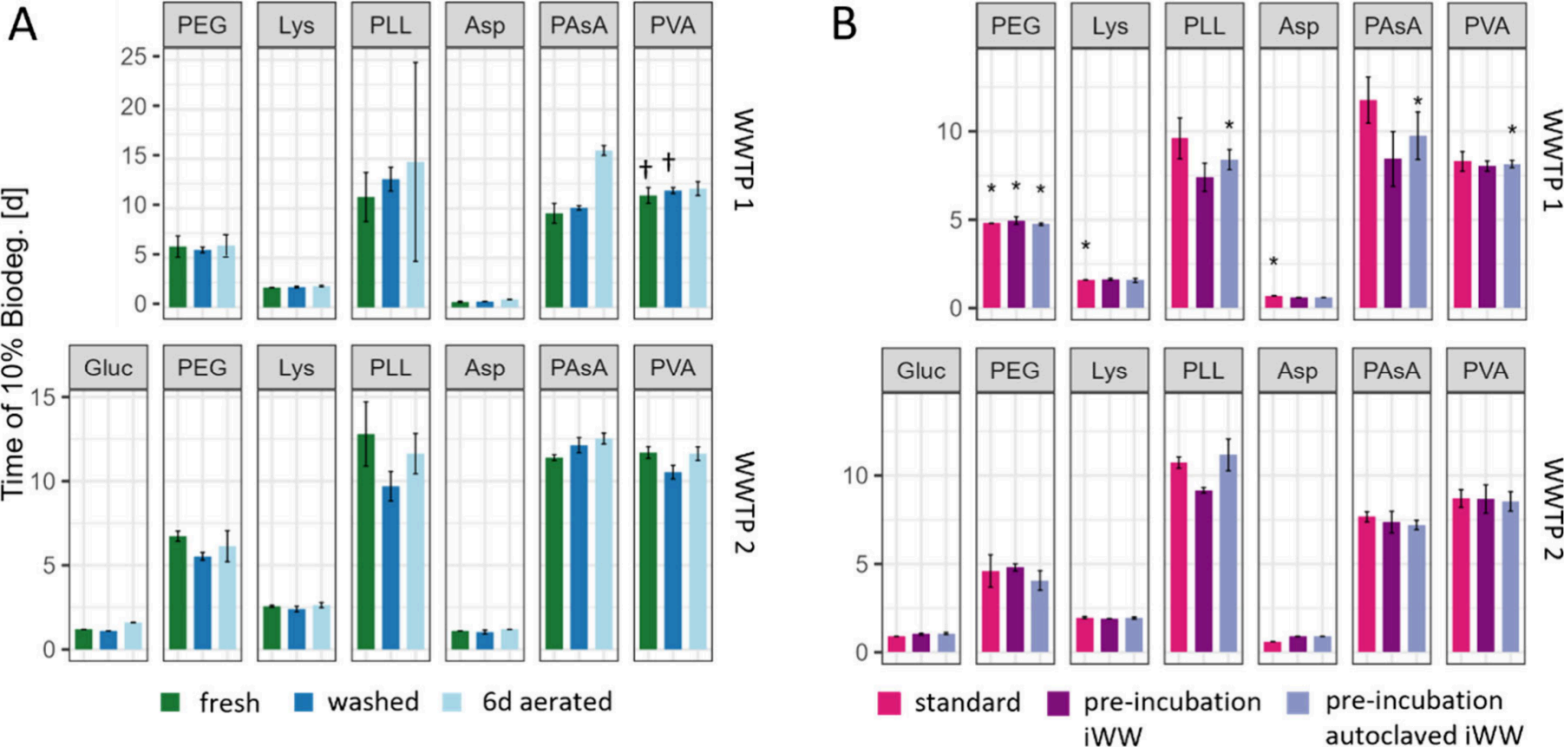
(A) Effect of inoculum washing and aeration (6 days) on WSP biodegradation. Times to reach 10% biodegradation were calculated based on theoretical CO_2_ production (ThCO_2_) using the BSBdigi-CO_2_ system for wastewater treatment plant (WWTP) 1 inocula and based on theoretical O_2_ demand (ThOD) using the OxiTop system for WWTP2 inocula. Error bars represent standard deviations of triplicate (WWTP2) and ranges of duplicates (WWTP1). Daggers (†) indicate incubations were lag phases were derived from linear interpolation due to data gaps caused by software malfunction (Figure S13). For WWTP1/PEG/6d aerated, a range is given that was visually determined (Figure S14). (B) Effect of Preincubation with filter-sterilized influent wastewater (iWW) on WSP biodegradation. Times to reach 10% biodegradation were calculated based on ThOD and measured O_2_ consumption using the OxiTop system for WWTP1 and 2. Error bars represent standard deviations of triplicates where not indicated differently (*, *n* = 2). Gluc: glucose, PEG: poly(ethylene glycol), PVA: poly(vinyl alcohol), Lys: lysine, PLL: ε-poly(l-lysine), Asp: aspartic acid, PAsA: poly(aspartic acid).

To investigate how washing and aeration of the microbial inocula affects WSP biodegradation, we incubated WSPs and low-molecular-weight substrates with inocula subjected to different pretreatments (Figure 2A and Figures S10A, S11, and S12). Untreated fresh inocula were used to maintain microbial community composition and extracellular enzymes abundance as close as possible to WWTP conditions. In parallel, we used inocula that were either washed or aerated (for 6 days). For WWTP1, we assessed biodegradation by simultaneously quantifying O_2_ consumption and CO_2_ production using the BSBdigi system; we conducted three separate experiments (i.e., one for lysine and PLL, one for aspartic acid and PAsA, and one for PVA and PEG). For WWTP2, we assessed biodegradation by quantifying O_2_ consumption using the OxiTop system in one experiment.

The effect of washing and aeration on background respiration was assessed using blank incubations without a substrate (Table S3). For WWTP1, aeration reduced average O_2_ consumption from 5.4 to 3.2 mg and average CO_2_ production from 8.9 to 6.2 mg. For WWTP2, aeration reduced average O_2_ consumption from 6.6 mg to 4.8 mg. For both WWTPs, washing had a smaller effect than aeration, with reductions in O_2_ consumption and CO_2_ production of less than 10%. Notably, all respirometric background signals were much lower than the signals generated by the biodegradation of the test substances. For example, the theoretical O_2_ consumption and CO_2_ production for glucose at the applied concentration (at 100% biodegradation) is 26.75 mg and 36.75 mg, respectively. We concluded that reducing background respiration through washing and aeration of the inocula is not needed for experiments with substrates biodegrading at similar rates as the substrates studied herein and for wastewaters with similar background respirations.

Regarding WSP biodegradation, the impact of washing and aeration differed between the two WWTPs. For WWTP2, washing and aeration had no substantial effect on the biodegradation dynamics of the studied substances (Figure 2 and Figures S10A and S11). For WWTP1, washing also had no substantial effect (Figure 2 and Figures S10A and S12). However, aerating the microbial inoculum from WWTP1 increased the lag phase of PAsA biodegradation 1.7-fold relative to the fresh inoculum. A repetition of this experiment in triplicate confirmed this effect (aeration increased lag phase 1.6-fold, Figure S15). For PLL, aeration resulted in an increased lag-phase variability between duplicates. When repeating this experiment in triplicate, we did not observe substantial differences between fresh and aerated sludge (Figure S15). For the other tested substrates (including the reference substrates), no effect of aeration was observed, based on which we concluded that the general microbial activity was not affected by aeration and washing. Taken together, we found that extended aeration can influence WSP biodegradation dynamics and that reproducible testing is possible without washing and aeration of the inoculum. Based on these results and acknowledging that further validation of these findings is required, we propose to avoid inoculum pretreatment for WSP biodegradation testing.

In an attempt to explain the above-described effect of inoculum aeration on the biodegradation dynamics of PAsA, we quantified the general activity of extracellular peptidases of the differently pretreated inocula. In brief, we produced extracellular filtrates by centrifugation and sterile-filtration from fresh, washed, and aerated inocula used in respirometric experiments and compared the peptidase activity using an assay based on fluorogenic casein.^36,49^ For WWTP1, aeration reduced extracellular peptidase activity by a factor of 0.76 (*p* = 0.0006, Figure S16). This observation might be explained by the inactivation or degradation of extracellular peptidases that hydrolyze both PAsA and casein–resulting in an increased lag phase of PAsA biodegradation and a lower peptidase activity measured with the casein-based assay. For WWTP2, aeration did not significantly reduce extracellular peptidase activity (*p* > 0.05), but inoculum washing did by a factor of 0.54 (*p* = 0.003). As inoculum washing did not affect WSP biodegradation, we concluded that peptidases whose activity was reduced by washing either played no role in WSP biodegradation or were rapidly replenished by microorganisms during the biodegradation experiment.

To investigate the effects of sludge washing and aeration on the microbial community composition, we conducted a 16S rRNA gene amplicon sequencing-based community analysis of the fresh, washed, and six-day-aerated sludge samples from WWTP1 and 2 used in the biodegradation tests. The treatments did not lead to significant differences in Shannon diversity and species richness (Chao 1) within or between treatments (Figure S17A/C). When the effect of the treatments on community composition (Aitchison distance) was compared, aerated sludge samples clustered separately from fresh and washed sludge samples for WWTP1 (Figure S17B). When testing the treatment effect on microbial community structure statistically (PERMANOVA), the difference was nonsignificant (*p*-value = 0.098). Nonetheless, the separate clustering of aerated samples motivates future research into microorganisms that are affected by aeration and might play a role in PAsA biodegradation.

### Effect of WSP Preincubation with Filter-Sterilized Wastewater on WSP Biodegradation

To investigate if extracellular enzymes in sewer systems break down the tested WSPs, we incubated the WSPs with filter-sterilized raw wastewater prior to biodegradation experiments (Figure 2B and Figures S18 and S19). Biodegradation was quantified for both WWTPs by quantifying O_2_ consumption using the OxiTop system. For WWTP1, we ran three separate experiments (i.e., one for lysine and PLL, one for aspartic acid and PAsA, and one for PVA and PEG). For WWTP2, we ran all incubations in one experiment.

We demonstrated the presence of active peptidases in filter-sterilized wastewater using the fluorogenic peptidase assay described above (Figure S20). Peptidase activities in filter-sterilized wastewater were higher for both WWTPs compared to filtrates from the aeration tanks of the respective WWTP (Figure S16). Furthermore, the results showed similar activities (<10% variation) among filtrates from the three inoculum sampling days at WWTP1 and confirmed the absence of peptidase activity in autoclaved filtrates (Figure S20).

Regarding WSP biodegradation, preincubation with filter-sterilized wastewater had minor effects on biodegradation dynamics (Figures S18 and S19). Notably, for WWTP2, preincubation with filter-sterilized wastewater slightly reduced the lag-phase during PLL biodegradation compared to the standard protocol and the autoclaved control (Figure 2B). For WWTP1, preincubation with active filtrates reduced lag phases for both PLL and PAsA. Here, lag phases of both poly(amino acids) were also reduced, albeit to a lesser extent, upon preincubation with autoclaved controls, suggesting a potential abiotic contribution (e.g., effect of adsorption of WSP to wastewater components) to the observed effect. For WWTP2, PAsA biodegradation remained unaffected by preincubation. Importantly, the observed reductions in the lag phases of PLL and PAsA biodegradation were not larger than the defined threshold of 1.5-fold. Preincubation had no effect on the biodegradation dynamics of the low-molecular-weight reference substrates PEG and PVA for both WWTPs.

### WSP Biodegradation at Different Concentrations and Effect of Pre-exposure

**Figure 3:**
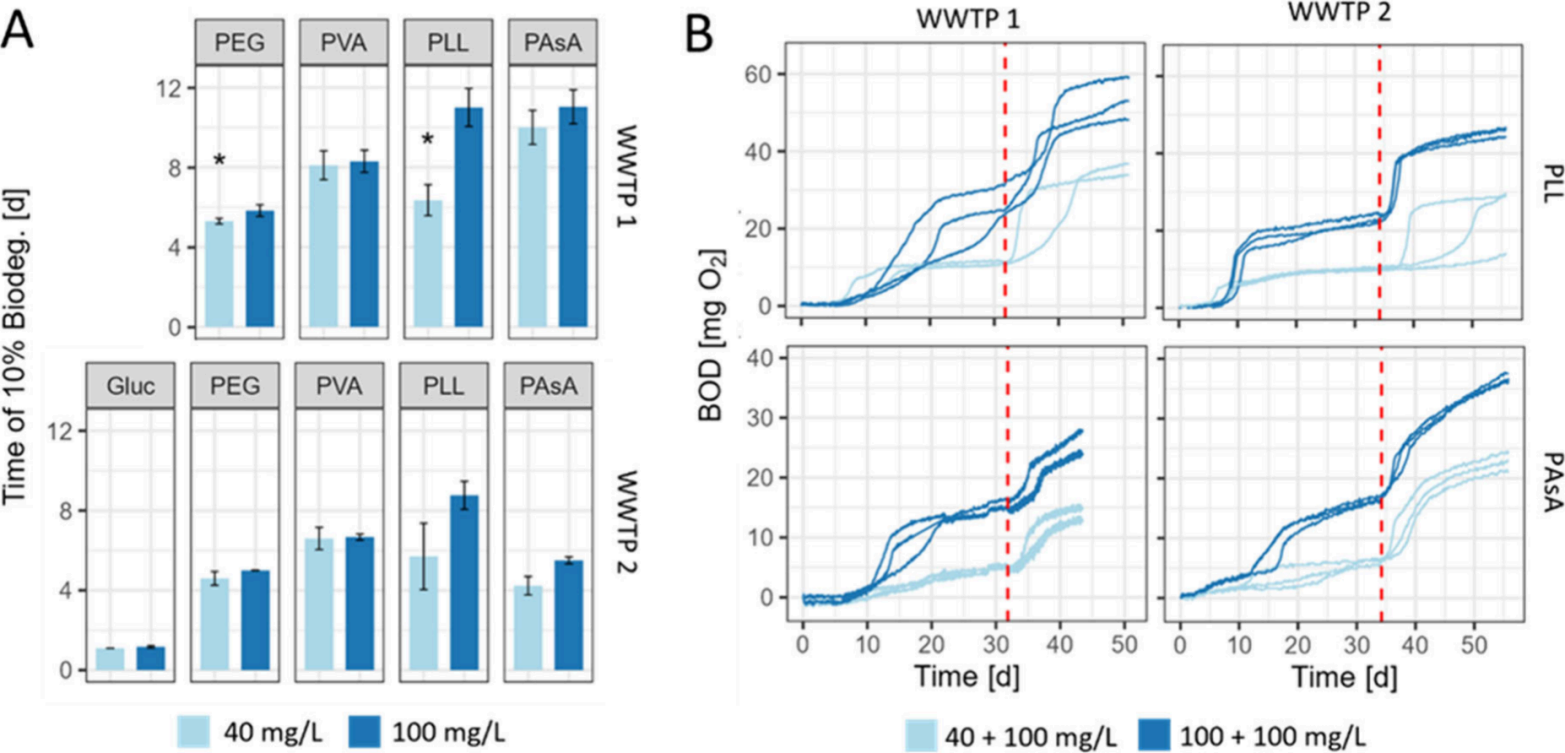
WSP biodegradation at different concentrations and after pre-exposure. (A) Times to reach 10% biodegradation were calculated based on theoretical O_2_ demand (ThOD) and measured O_2_ consumption using the OxiTop system. Error bars represent standard deviations of triplicates or ranges of duplicates, where indicated with an asterisk (*). (B) Biodegradation curves of blank-corrected biological oxygen demand (BOD) for wastewater treatment plants (WWTP) 1 and 2. Red vertical lines mark the time points, at which test substrate was added a second time (i.e., respike). Gluc: glucose, PEG: poly(ethylene glycol), PVA: poly(vinyl alcohol), PLL: ε-poly(l-lysine), PAsA: poly(aspartic acid).

To investigate the effect of WSP concentration on their biodegradation, we conducted experiments at two different concentrations: 100 mg/L, following the OECD 301 testing guideline, and 40 mg/L, which was selected as the lowest concentration that results in sufficient signal-to-noise ratios for all substrates and both test systems (Figure 3A and Figures S21A and S22A). Biodegradation was assessed via O_2_ consumption using the OxiTop system. For WWTP1, we ran each WSP in a separate experiment (i.e., a separate set of incubations for PEG, PVA, PLL, and PAsA). For WWTP2, we ran all incubations in one experiment.

For all tested substrates and WWTPs, the selected starting concentrations did not impact the final biodegradation extent (Figures S21A and S22A). However, biodegradation lag phases differed between the two concentrations for some WSPs (Figure 3A). For PLL, we found substantially shorter lag phases in experiments conducted with 40 mg/L compared with 100 mg/L, with reductions below a factor of 0.66 for both WWTPs. For WWTP2, the difference was, however, not statistically significant (*p* = 0.07). For WWTP1, repetition of the experiment in triplicates confirmed a smaller lag phase (0.66 fold) at 40 mg/L compared to 100 mg/L (*p* = 0.003, Figure S23). This effect might be ascribed to concentration-dependent antimicrobial properties of PLL.^40^ To test if PLL had an inhibitory effect on microbial activity, we assessed glucose biodegradation by inocula from both WWTPs in the presence and absence of PLL (Figure S24). This test showed that the presence of PLL (100 mg/L) delayed the onset of glucose biodegradation by approximately 4 days and motivated a systematic investigation of this effect (e.g., concentration dependence) in future work. We note that such inhibitory effects limit biodegradation comparisons (at high concentrations) between substances, which was, however, not the aim of this study that focused on comparisons between protocol variations. For PAsA, the effect of concentration pointed in the same direction but was subtle. No effect of concentration on biodegradation was observed for PEG, PVA, and glucose.

**Figure 4:**
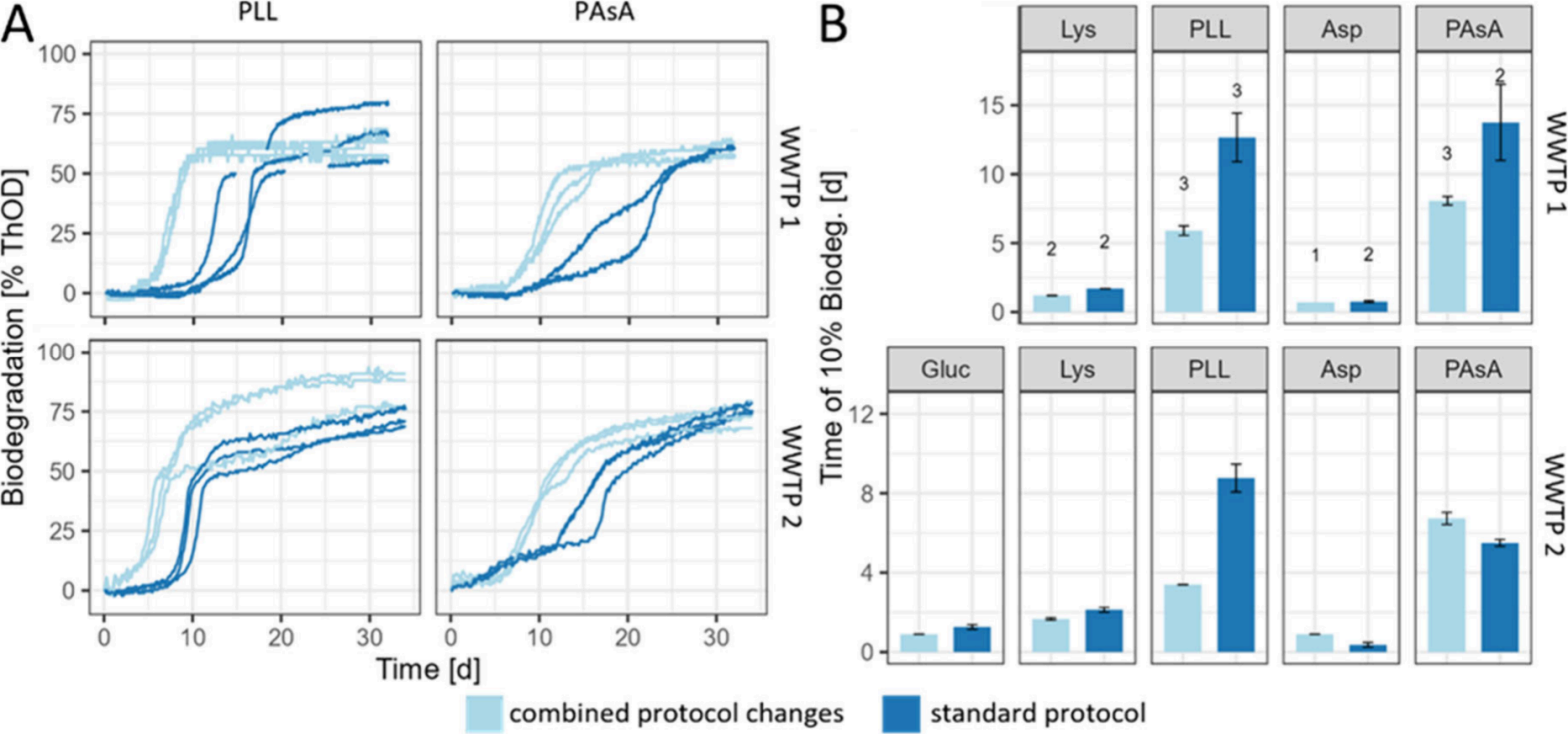
Combined effect of inoculum aeration, preincubation with filter-sterilized wastewater, and WSP concentration on WSP biodegradation. (A) Biodegradation curves calculated based on theoretical O_2_ demand (ThOD) and measured O_2_ consumption using the OxiTop system for wastewater treatment plant (WWTP) 1 and 2 inocula. (B) Times to reach 10% biodegradation. Error bars represent standard deviations of triplicates, where not indicated differently (*n* = #, indicated above each bar). Gluc: glucose; Lys: lysine; PLL: ε-poly(l-lysine); Asp: aspartic acid; PAsA: poly(aspartic acid).

To assess how pre-exposure of microorganisms to the test substance affects biodegradation, we prolonged the experiments described above. After reaching a plateau, the test substance was repspiked (second spike at 100 mg/L; Figures 4B and Figures S21B and S22B). A quantitative evaluation (e.g., calculation of lag phases) of the respike experiment was not conducted, as in some cases the originally spiked substances were not completely degraded (no complete plateau) when spiking the second time. However, a qualitative curve comparison revealed, that almost all tested combinations of substances and inocula (exceptions discussed below), showed a rapid onset in biodegradation shortly (i.e., within 4 days) after the substrate respike. The shortening of the lag phase upon adaptation can be an indicator of the induction of existing metabolic pathway.^24^ For example, PAsA that was incubated with pre-exposed microorganisms from WWTP1 exhibited a significantly earlier onset in biodegradation (within 1–3 days) upon respiking, compared to the initial experiment lag phases of 10–12 days. This adaptation occurred regardless of whether the inoculum was pre-exposed to 40 or 100 mg/L PAsA during the first experiment, indicating adaptation of the microbial metabolism to the test substance.

One exception was the biodegradation of PVA by microorganisms from WWTP1. Upon respiking PVA, biodegradation accelerated in only one replicate, while two others exhibited a more continuous biodegradation (Figure S21B). Glucose biodegradation (WWTP2) remained consistent during the first and second spikes. ε-poly(l-lysine) incubated with inocula initially exposed to 40 mg/L showed a high variability in biodegradation after the second spike (Figure 3B), likely due to starvation and (partial) loss of the required microbial or enzymatic activity. This is supported by experiments with WWTP1 inocula, where spiking WSPs to starved inocula (that have been incubated without substrate during the first part of the experiment; Figure S21B) substantially delayed the onset of biodegradation for all polymers compared with inocula treated according to the standard protocol.

### Combined Effects of Inoculum Aeration, Preincubation with Untreated Wastewater Extract, and Concentration on WSP Biodegradation

Building on the experiments described above, we assessed the combined effects of these protocol variations with a primary focus on PAsA and PLL (Figure 4). For PLL, the most relevant combination of variations included (i) the use of fresh microbial inoculum, (ii) preincubation with filter-sterilized wastewater, and (iii) a lower WSP concentration (i.e., 40 mg/L). For PAsA, the adapted protocol mirrored that of PLL, excluding the lower concentration due to its limited effect on the previous test outcome (see above, Figure 3A and the corresponding text). For comparison, we conducted parallel experiments according to standard protocol (i.e., 3-day aeration, no WSP preincubation with filter-sterilized wastewater, and a substrate concentration of 100 mg/L). All incubations were run in one experiment for both WWTPs, measuring biodegradation via O_2_ consumption using the OxiTop system.

While no substantial differences in biodegradation curves were observed for the low-molecular-weight reference substrates between the adapted and standard protocols (Figure 4 and Figures S25 and S26), biodegradation curves of PLL and PAsA were substantially different between the two protocols. For PLL, the time to reach 10% biodegradation decreased by a factor of 0.46 (p = 0.04) for WWTP1 and by a factor of 0.38 (*p* = 0.006) for WWTP2 under the combined protocol variations (Figure 4). Also, for PASA, biodegradation by microorganisms from both WWTPs was faster under the adapted protocol conditions. For WWTP1 the lag-phase of PAsA decreased by a factor of 0.58. Repeating this experiment in triplicates confirmed a reduction by a factor of 0.56 (*p* = 0.023, Figure S27). For WWTP2 no substantial effects of the combined protocol variation on the lag phase was observed, but the time to reach 50% biodegradation was reduced by a factor of 0.68 (*p* = 0.014) in response to the protocol adaptations.

## Environmental Implications

Our study showed that protocol variations, such as avoiding inoculum aeration, preincubation of WSPs with wastewater enzyme extracts, or lowering WSP concentrations, can influence the results of WSP biodegradation testing based on respirometric laboratory experiments. While individual variations generally showed small effects and effects sometimes varied between the microbial inocula derived from the two tested wastewater treatment plants, the combination of specific protocol variations substantially accelerated the biodegradation of the tested poly(amino acids). Importantly, these protocol variations had no substantial effect on the biodegradation dynamics of the low-molecular-weight reference substrates tested herein, suggesting that the effects are indeed specific for polymers and probably linked to the involvement of extracellular enzymes such as peptidases for poly(amino acids). The tested variations might thus be the first step in the process of adapting biodegradation testing protocols from small molecules to polymers. Future studies should combine WSP biodegradation tests with specific enzyme activity assays to identify the links between WSP biodegradation and enzyme activity. Such links would enable fast assessments of the potential of a microbial sample for WSP biodegradation.

The herein reported biodegradation of PLL, which has previously not been tested with respirometric methods, is a promising result for the development of biodegradable WSPs based on lysine, and possibly other cationic amino acids. The promising biodegradation dynamics observed for all four tested WSPs call for research systematically linking chemical structure variations of these WSPs with biodegradability. The observed effect of preexposing wastewater microorganisms to the tested WSPs highlights that their potential to biodegrade WSPs can change during the testing incubations. Future work should identify the concentration thresholds of such substrate adaptations and assess the transferability of results from laboratory batch incubations (typically conducted at high substance concentrations and low microorganism concentrations) to realistic WWTP conditions (i.e., lower substance concentrations and higher microorganism concentrations).

While our study highlights the importance of laboratory testing that does not require expensive materials or highly specialized equipment and allows a high sample throughput, more advanced methods are required for in-depth studies of promising WSPs. For example, carbon-isotope-labeled WSPs in respirometric methods distinguish between WSP mineralization and the mineralization of matrix components and thus enable rigorous carbon balances.^52−54,56^ Additionally, the development of methods to quantify and characterize the nonmineralized WSP fraction at specific times during biodegradation tests (and to thereby learn more about the biodegradation process) is essential.^57,58^ These approaches could complement laboratory experiments and contribute to a tiered biodegradation testing scheme that paves our way toward nonpersistent WSPs.^8,59,60^

## Supporting Information Available

Texts S1–S4: Additional chemicals; elemental composition and MW determination; OECD 301 buffer composition; 16S rRNA gene sequencing; Figures S1–S27: Scheme of experiments; Illustration of respirometric systems; Extended biodegradation curves; O_2_ and CO_2_ derived biodegradation curves; Effect of acid spiking on the final CO_2_ production; Respirometric system comparison; Interday variability WWTP1; Interday variability WWTP2; Abiotic experiments; Single protocol adaptations for poly(amino acid)s; Effect of sludge washing and aeration on WSP biodegradation; Interpolation of data gaps; Visual determination of lag-phase; Repetition experiment for polyamino acids; Peptidase activity sludge inoculum; 16S rRNA community analysis; Effect of polymer preincubation with iWW; Peptidase activity of iWW; Polymer concentration and pre-exposure WWTP1; Polymer concentration and pre-exposure WWTP2; Repetition concentration dependence PLL; PLL inhibitory test; Combined protocol changes; Repetition experiments PAsA standard and combined protocol changes; Tables S1–S3: Substrate characteristics; WWTP characteristics; Background respiration (PDF)
