# Supplementary material for: Biodegradation of Water-Soluble Polymers by Wastewater Microorganisms: Challenging Laboratory Testing Protocols
Source: Environ Sci Technol. 2024 Aug 12;58(34):15246–56. doi: 10.1021/acs.est.4c05808 (PMC11360367; doi:10.1021/acs.est.4c05808)
Supplement: Supplementary file 1 — es4c05808_si_001.pdf [file es4c05808_si_001.pdf]

Supporting Information

Biodegradation of water-soluble polymers by wastewater microorganisms: challenging laboratory testing protocols

Aaron Kintzi[1,2], Soumya Daturpalli[3], Glauco Battagliarin[3], and Michael Zumstein[*,1]

[1]Division of Environmental Geosciences, Centre for Microbiology and Environmental Systems Science, University of Vienna, Josef-Holaubek-Platz 2, 1090 Vienna, Austria

[2]Doctoral School in Microbiology and Environmental Science, University of Vienna, 1090 Vienna, Austria

[3]BASF SE,67056 Ludwigshafen am Rhein, Germany

[*]To whom correspondence should be addressed:

E-mail: michael.zumstein@univie.ac.at

This PDF file includes:

Number of pages: 38

Number of texts: 4

Number of figures: 27

Number of tables: 3

Text elements:

Text S1. Additional Chemicals.

Text S2. Analysis of elemental composition and molecular weight determination.

Text S3. Composition of OECD 301 buffer.

Text S4. Community profiling using 16S rRNA gene amplicon sequencing.

Figures:

Figure S1. Scheme of conducted biodegradation experiments and the corresponding main figures.

Figure S2. Illustration of the respirometric systems OxiTop® and BSBdigi-CO_2®.

Figure S3. Biodegradation of WSPs by microorganisms from two wastewater treatment plants (WWTPs).

Figure S4. Comparison of biodegradation curves derived from O_2 consumption and CO_2 production using the BSBdigi-CO_2® system.

Figure S5. Effect of acid spiking on the final CO_2 production of glucose biodegradation using the BSBdigi-CO_2® system and inoculum from wastewater treatment plant (WWTP) 1.

Figure S6. Comparison of the OxiTop® and BSBdigi-CO_2® respirometric systems using the same microbial inoculum from wastewater treatment plant (WWTP) 1.

Figure S7. Inter-day variability between biodegradation experiments using inoculum from wastewater treatment plant (WWTP) 1.

Figure S8. Inter-day variability between biodegradation experiments using inoculum from wastewater treatment plant (WWTP) 2.

Figure S9. Abiotic control experiment for WSPs and low-molecular weight reference compounds.

Figure S10. Effect of different protocol adaptations on poly(amino acids) biodegradation.

Figure S11. Effect of inoculum washing and aeration (6 days) on WSP biodegradation using inoculum from wastewater treatment plant (WWTP) 2.

Figure S12. Effect of inoculum washing and aeration (6 days) on WSP biodegradation using inoculum from wastewater treatment plant (WWTP) 1.

Figure S13. Linear interpolation for data gaps caused by BSBdigi-CO_2® software malfunctioning.

Figure S14. Visual determination of the time to reach 10% biodegradation for PEG using 6-day aerated inoculum.

Figure S15. Repetition: Comparison of PAsA and PLL biodegradation with fresh and 6-day aerated inoculum from wastewater treatment plant (WWTP) 1.

Figure S16. Effect of inoculum washing and aeration (6 days) on peptidase activity measured with EnzChek Protease Assay Kit (Thermo Fisher, E6638).

Figure S17. 16S rRNA community analysis for sludge samples from wastewater treatment plant (WWTP) 1 and 2.

Figure S18. Effect of pre-incubation with filter-sterilized influent wastewater (iWW) on WSP biodegradation using inoculum from wastewater treatment plant (WWTP) 1.

Figure S19. Effect of pre-incubation with filter-sterilized influent wastewater (iWW) on WSP biodegradation using inoculum from wastewater treatment plant (WWTP) 2.

Figure S20. Peptidase activity of filter-sterilized influent wastewater (iWW) measured with EnzChek Protease Assay Kit (Thermo Fisher, E6638).

Figure S21. WSP biodegradation at different concentrations and pre-exposure using inoculum from wastewater treatment plant (WWTP) 1.

Figure S22. WSP biodegradation at different concentrations and pre-exposure using inoculum from wastewater treatment plant (WWTP) 2.

Figure S23. Repetition: PLL biodegradation at 40 and 100 mg/L with inoculum from wastewater treatment plant (WWTP) 1.

Figure S24. PLL inhibitory test with sludge from both wastewater treatment plants (WWTP)s.

Figure S25. Combined effects of sludge aeration, pre-incubation with filter-sterilized influent wastewater (iWW), and concentration on biodegradation using inoculum from wastewater treatment plant (WWTP) 1.

Figure S26. Combined effects of sludge aeration, pre-incubation with filter-sterilized influent wastewater (iWW), and concentration on biodegradation using inoculum from wastewater treatment plant (WWTP) 2.

Figure S27. Repetition: PAsA biodegradation under standard protocol and combined protocol variations conditions for wastewater treatment plant (WWTP) 1.

Tables:

Table S1. Substrate characteristics.

Table S2. WWTP characteristics.

Table S3. Effect of inoculum washing and aeration (6 days) on background respiration in blank samples.

Text S1. Additional Chemicals.

Amberlite HPR1100 Na-ion exchange resin (91973), copper (II) sulphate pentahydrate (209198), dipotassium hydrogen orthophosphate (P8281), N-allylthiourea (108804), and potassium dihydrogen orthophosphate (P0662) were purchased from Sigma-Aldrich. Ammonium chloride (1.01143), disodium hydrogen orthophosphate dihydrate (1.06580), calcium chloride (1.02378), hydrochloric acid (Titripur, 1.09057), iron (III) chloride hexahydrate (1.03943), magnesium sulphate heptahydrate (1.05886), potassium hydroxide solution (Titripur, 1.09108), potassium hydrogen phthalate (Certipur, 1.02400), Sodium carbonate (Certipur, 1.02405) were purchased from Merck. Ultrapure water was obtained from a water purification system (0.071 µS/cm, Elga Veolia, PURELAB Chorus).

Text S2. Analysis of elemental composition and molecular weight determination.

To determine the carbon (as CO_2), hydrogen (as H_2O), and nitrogen (as N_2) content of the polymers, 1-10 mg were combusted in a helium/oxygen atmosphere and measured using an elemental analyser (Elementar, Vario EL Cube). To determine the oxygen content, 1-10 mg were pyrolyzed and reduced in a forming gas atmosphere on a soot contact. The converted oxygen to carbon monoxide (CO), was quantified by IR spectrometry using an elemental analyser (Elementar, Rapid OXY cube). The molecular weight (MW) of non-commercial WSPs was determined by Size Exclusion Chromatography using a TKSgelG3000PWxl column with a flow rate of 0.5 mL/min column temperature 35°C, injection volume of 100 µL, and polymer concentration: 1.5 mg/mL. The mobile phase consisted of 0.01 mol/L phosphate buffer (10 mM Na_2HPO_4 + 1.8 mM KH_2PO_4 + 2.7 mM KCl + 137 mM NaCl) at pH 7.4 containing 0.01 M NaN_3. Polymers were dissolved in the mobile phase. Detection was performed using a DRI Agilent 1100 and UV Agilent 1100 VWD detector. Calibration was achieved using narrowly distributed Na-PAA standards covering molecular weights from M = 1,250 to M = 146,000 (PSS Polymer Standards Service GmbH; Mainz, Germany), and PAA standards with molecular weights of M = 1,770 and M = 900 g/mol (American Polymer Standards).

Text S3. Composition of OECD 301 buffer.

Biodegradation experiments were conducted in OECD buffer as described in OECD guideline 301 A[1] consisting of KH_2PO_4 (0.625 mM), K_2HPO_4 (1.249 mM), Na_2HPO_4*2 H_2O (1.877 mM), NH_4Cl (0.093 mM), $CaCl_2$ (0.248 mM), $MgSO_4$*7 H_2O (0.0913 mM) and $FeCl_3$*6 H_2O (0.0009 mM). The pH was adjusted to pH 7.4 using hydrochloric acid (HCl).

Text S4. Community profiling using 16S rRNA gene amplicon sequencing.

To study the effects of washing and aeration on the sludge microbial community, we collected three samples from WWTP 1 at three distinct timepoints and one sample for WWTP 2. We preserved 2 mL aliquots of each sludge type by freezing them in liquid nitrogen and storing them at -20°C until analysis.

DNA was extracted from sludge samples using the PowerSoil Pro extraction kit (Qiagen, Germany), and the V4 hypervariable region of the bacterial and archaeal 16S rRNA gene was amplified using primers 515F/806R [2,3] DNA extraction, sequencing, and raw data processing was performed at the Joint Microbiome Facility of the Medical University of Vienna and the University of Vienna (project ID JMF-2311-02) as described previously[4], and sequenced on a Illumina MiSeq (2 x 300bp). Amplicon pools were extracted from the raw sequencing data using the FASTQ workflow in BaseSpace (Illumina) with default parameters. Raw data processing was performed as described previously[4]. Demultiplexing was performed with the python package demultiplex[5] allowing one mismatch for barcodes and two mismatches for linkers and primers. Amplicon sequence variants (ASVs) were inferred using the DADA2 R package v1.42[6] applying the recommended workflow[7]. FASTQ reads 1 and 2 were trimmed at 220 nt and 150 nt with allowed expected errors of 2. ASV sequences were subsequently classified using DADA2 and the SILVA database SSU Ref NR 99 release 138.1[8,9] using a confidence threshold of 0.5. ASVs without classification or classified as eukaryotes, mitochondria, or chloroplasts, as well as well-known buffer contaminations were removed. After filtering, only samples with at least 1000 read pairs were kept for further analyses.

Downstream analyses were performed using R v4.3.2 and Bioconductor v3.16 packages SummarizedExperiment v1.32, SingleCellExperiment v1.24, TreeSummarizedExperiment v2.8[10], mia v1.8[11], vegan v2.6-4[12], phyloseq v1.44[13] , microbiome v1.22[14], microViz v0.10.8[15,16], and DESeq2 v1.42[17]. Alpha diversity (i.e., richness and diversity indexes) was calculated on rarified data (1253 reads/sample) using R packages vegan and mia. Beta diversity was calculated by performing a PCoA with Aitchison distance using R package microViz. The difference in per-group centroids was tested with a PERMANOVA on Aitchison distance using R packages vegan and microViz. The 16S rRNA gene amplicon sequencing data was deposited under the BioProject ID PRJNA1139083.

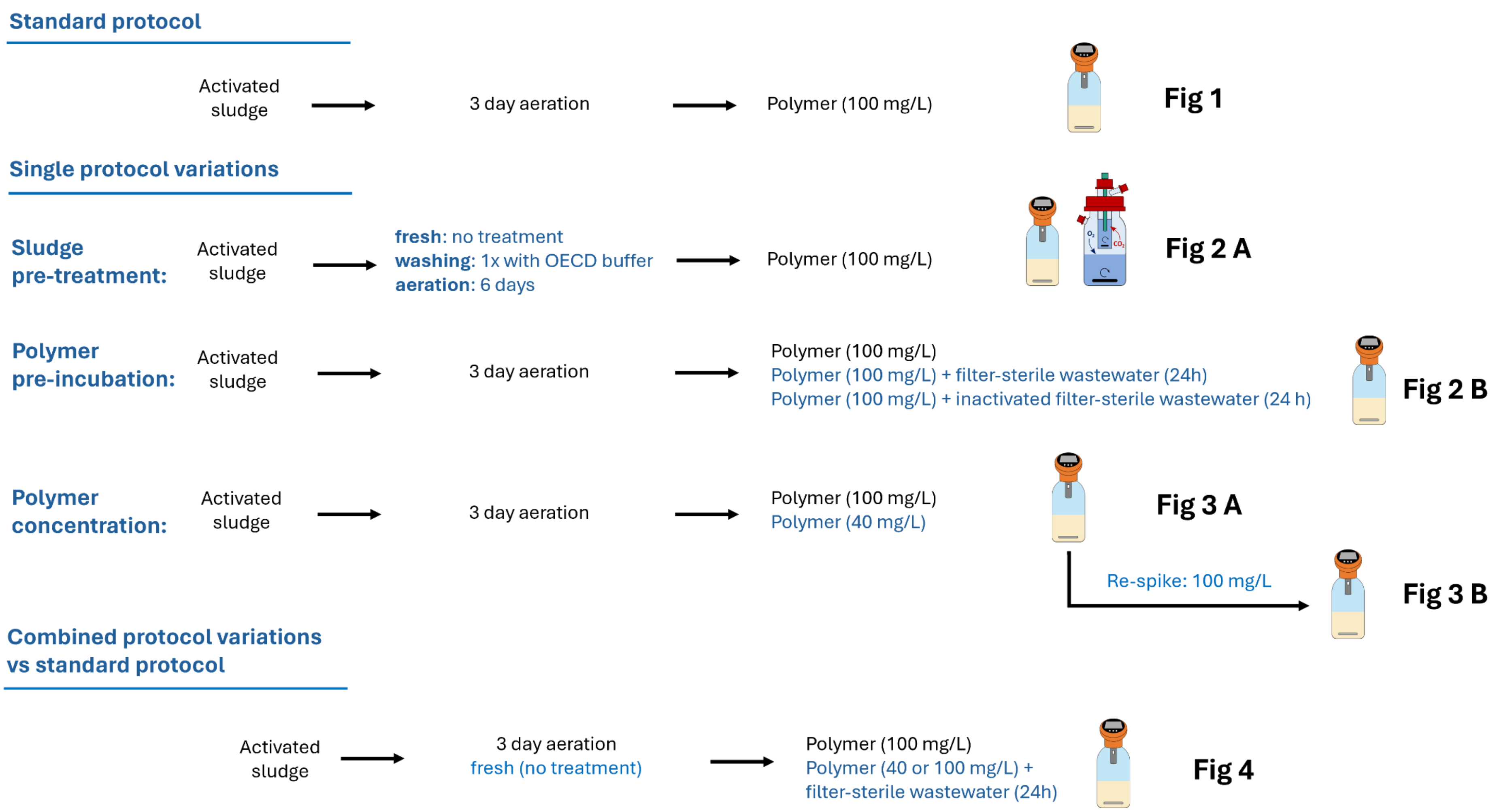

Figure S1. Scheme of conducted biodegradation experiments and the corresponding main figures.

.

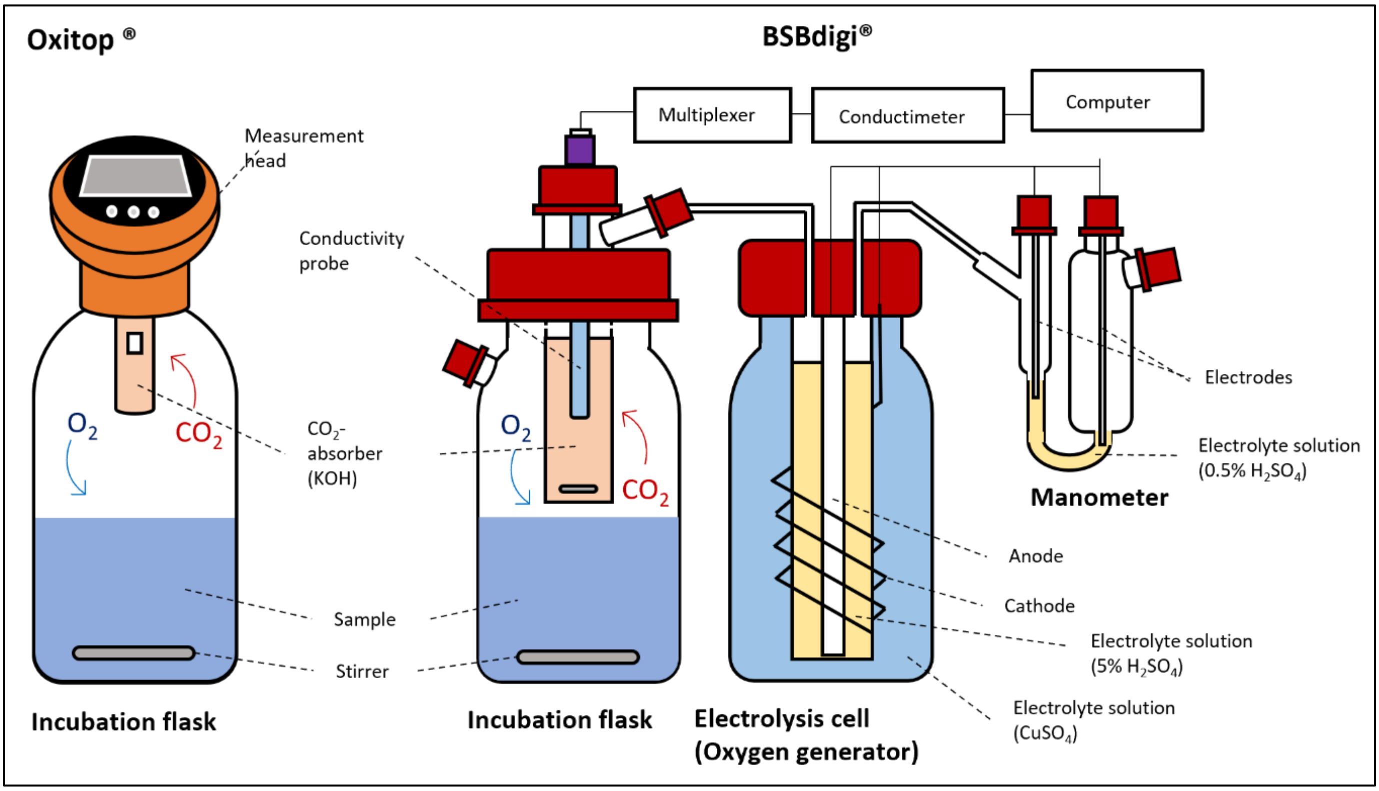

Figure S2: Illustration of the respirometric systems OxiTop® and BSBdigi-CO_2®.

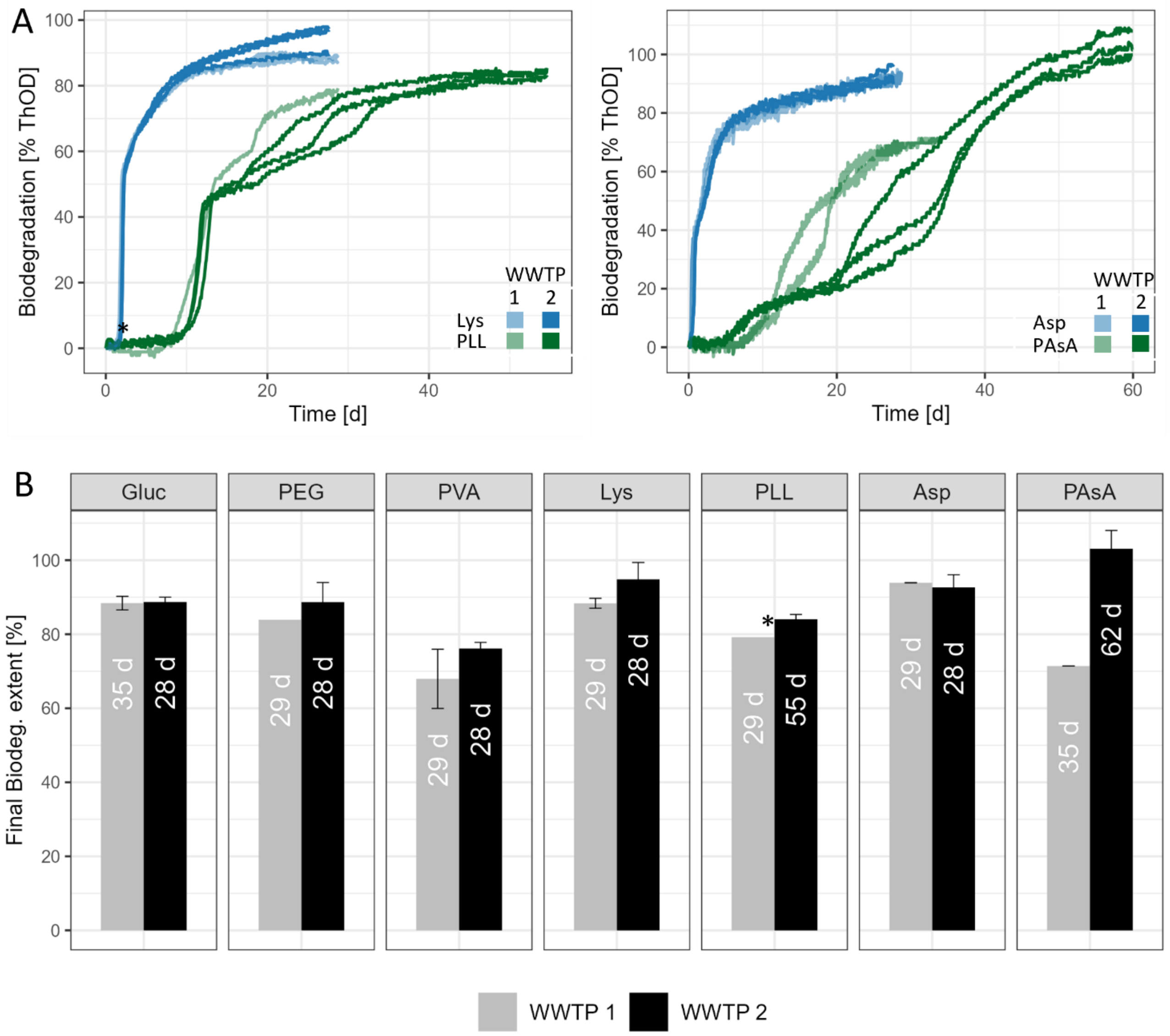

Figure S3. Biodegradation of WSPs by microorganisms from two wastewater treatment plants (WWTPs). **(A)** Extended biodegradation curves calculated based on theoretical O_2 demand (ThOD) and measured O_2 consumption during WSP incubation using the OxiTop® system. Asterisks (*) indicate incubations that had to be stopped at the indicated time point due to instrument malfunctioning. **(B)** Biodegradation extents at the end of the experiments. The duration of each experiment is indicated within the bars. Error bars represent, where not otherwise stated with an asterisk (*), standard deviations of triplicates and ranges of duplicates for WWTP 2 and WWTP 1, respectively. The data shown here is an extended version of the data shown in Figure 1. Gluc: glucose, PEG: poly(ethylene glycol), PVA: poly(vinyl alcohol), Lys: lysine, PLL: ε-poly(L-lysine), Asp: aspartic acid, PAsA: poly(aspartic acid).

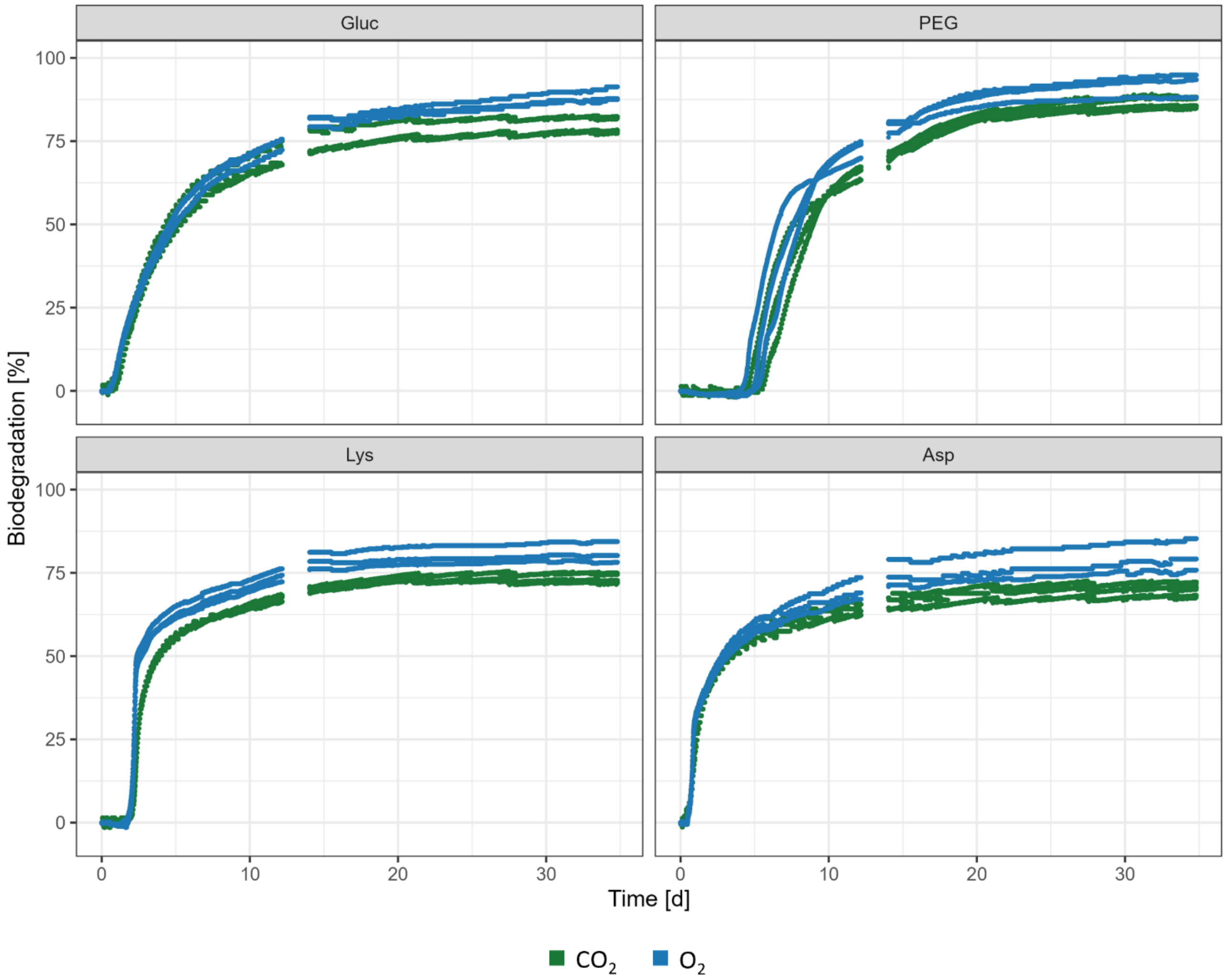

Figure S4. Comparison of biodegradation curves derived from O_2 consumption and CO_2 production using the BSBdigi-CO_2® system. Biodegradation curves were calculated based on theoretical O_2 demand (ThOD) and measured O_2 consumption and based on theoretical ($ThCO_2$) and measured CO_2 production, respectively. Data gap between days 12 and 14 was caused by instrument software malfunctioning. Gluc: glucose, PEG: poly(ethylene glycol), Lys: lysine, Asp: aspartic acid.

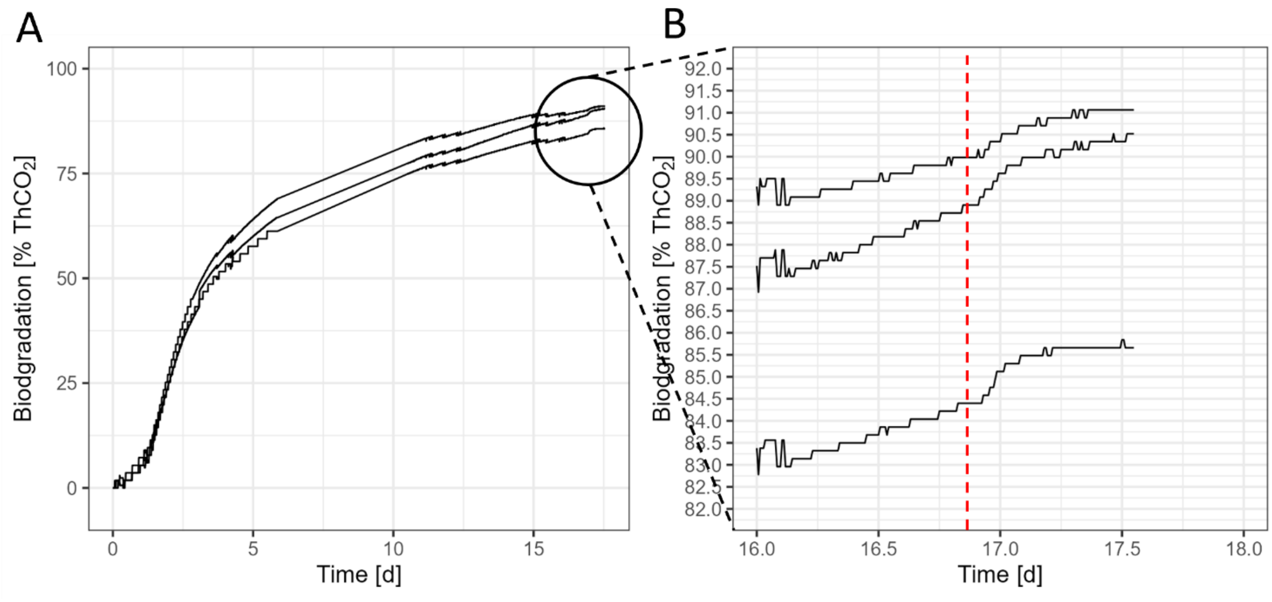

Figure S5. Effect of acid spiking on the final CO_2 production of glucose biodegradation using the BSBdigi-CO_2® system and inoculum from wastewater treatment plant (WWTP) 1. The red dashed line indicates the time of acid addition (1 mL of 1 M HCl to the incubation solution) through a septum using a gas tight syringe.

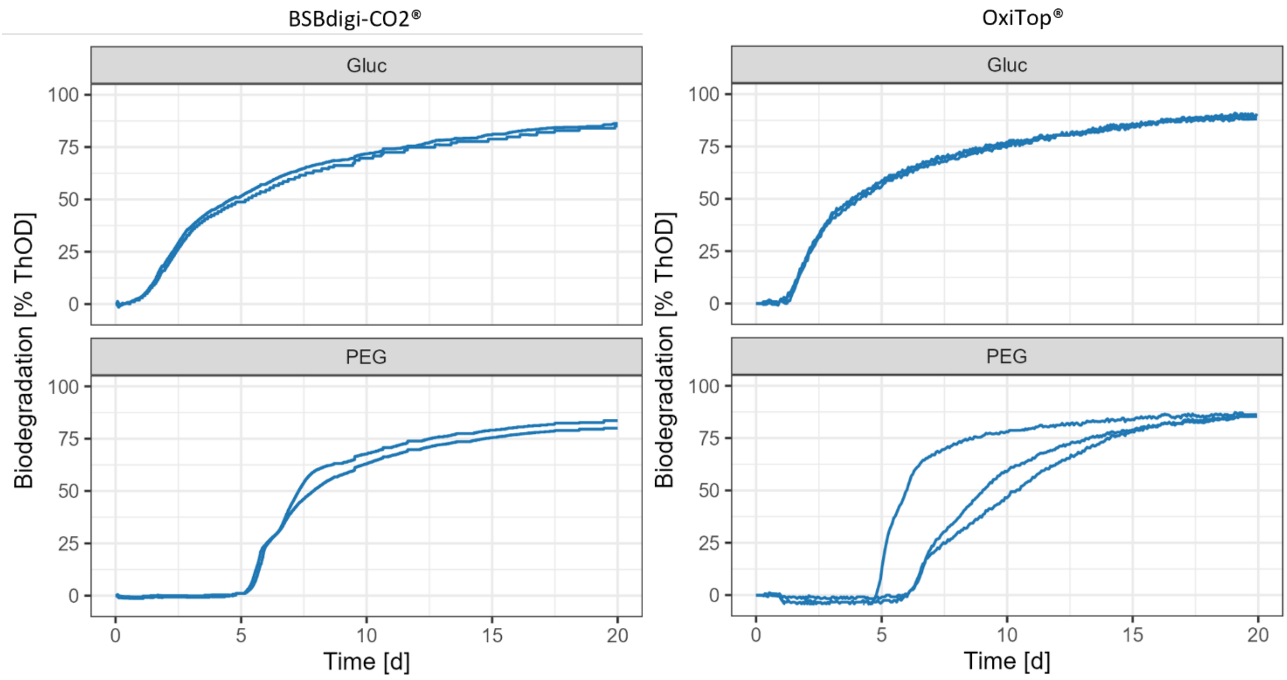

Figure S6: Comparison of the OxiTop® and BSBdigi-CO_2® respirometric systems using the same microbial inoculum from wastewater treatment plant (WWTP) 1. Biodegradation curves were calculated based on theoretical O_2 demand (ThOD) and measured O_2 consumption. Gluc: glucose, PEG: poly(ethylene glycol).

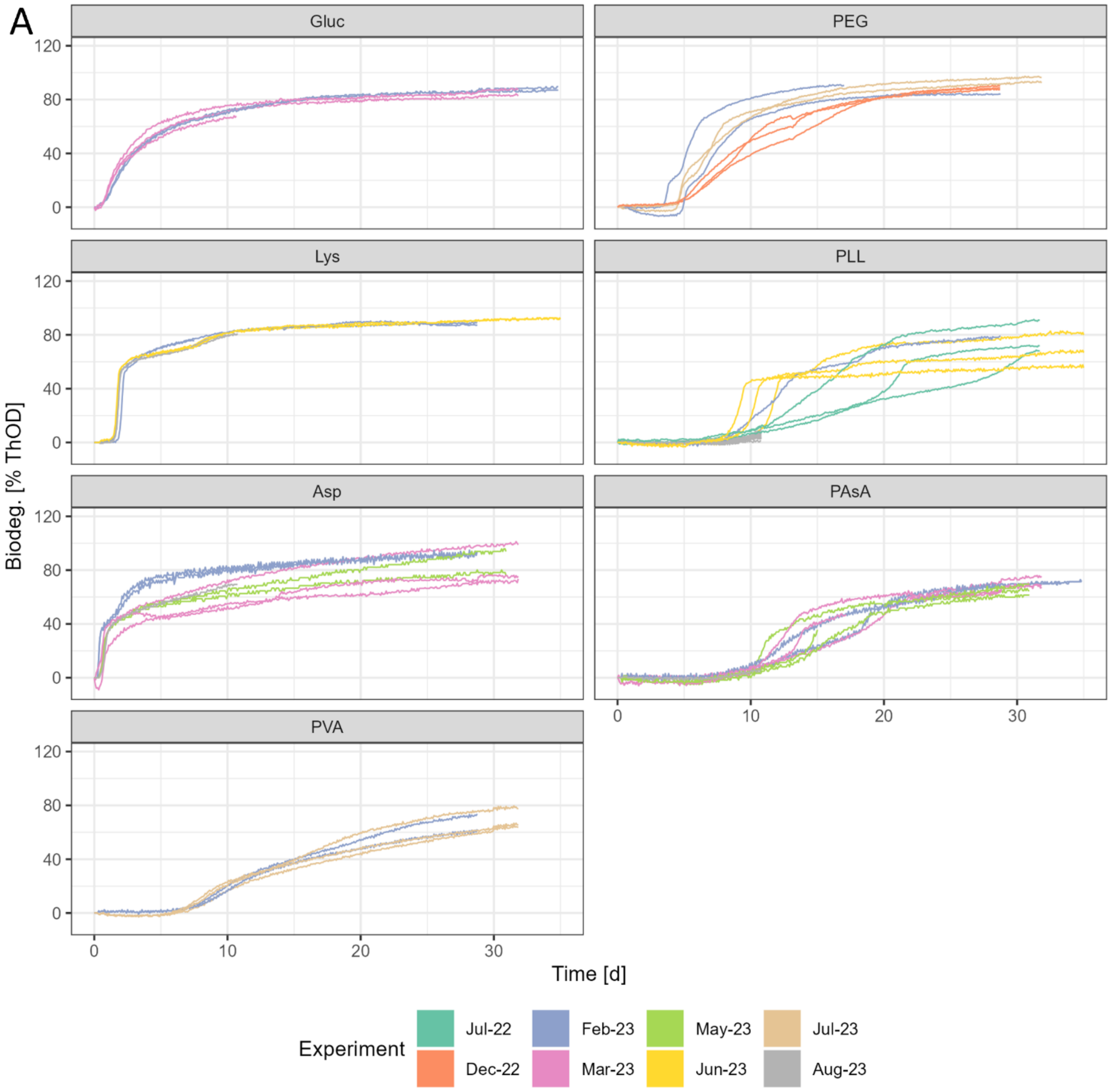

Figure S7; continued on next page

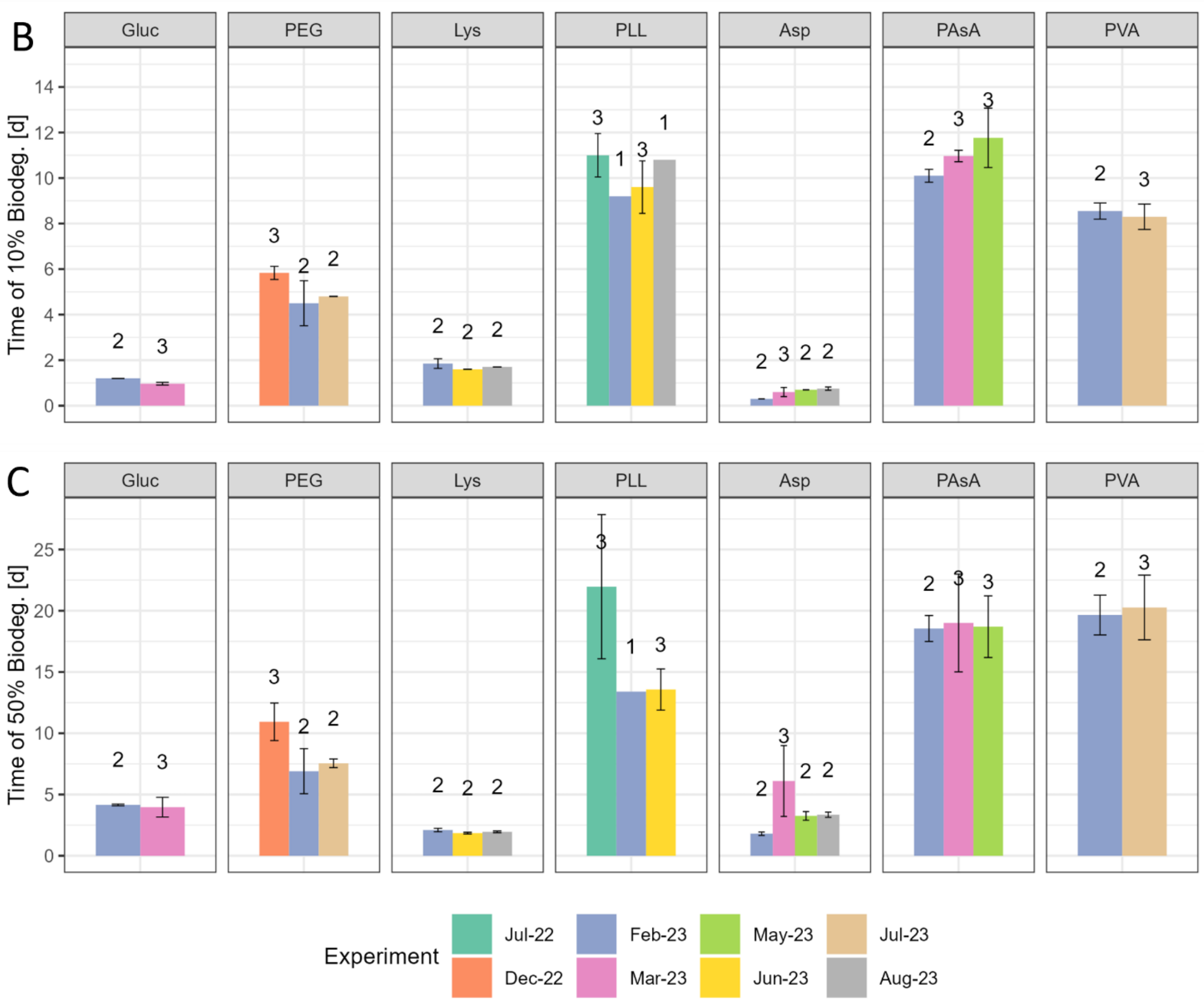

Figure S7. Inter-day variability between biodegradation experiments using inoculum from wastewater treatment plant (WWTP) 1. **(A)** Biodegradation curves calculated based on theoretical O_2 demand (ThOD) and measured O_2 consumption during WSP incubation using the OxiTop® system. **(B)** and **(C)** Times to reach 10% and 50% biodegradation, respectively. The data for February, March, June, and July 2023 is identical to the standard experimental data depicted in **Figure 1, 2B, 3,** and **4,** respectively. Gluc: glucose, PEG: poly(ethylene glycol), Lys: lysine, PLL: ε-poly(L-lysine), Asp: aspartic acid, PAsA: poly(aspartic acid), PVA: poly(vinyl alcohol).

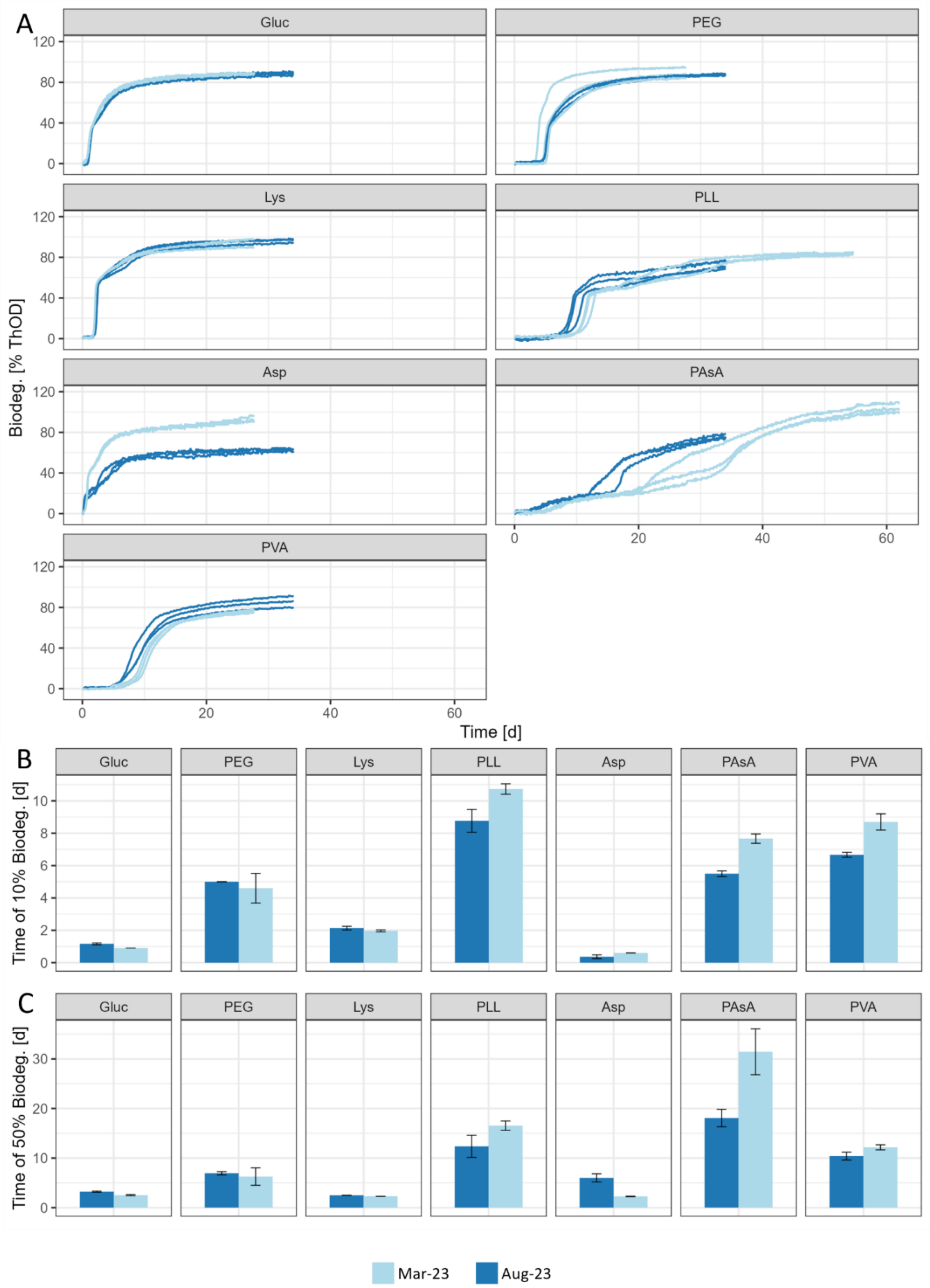

Figure S8. Inter-day variability between biodegradation experiments using inoculum from wastewater treatment plant (WWTP) 2. **(A)** Biodegradation curves calculated based on theoretical O_2 demand (ThOD)

and measured O_2 consumption during WSP incubation using the OxiTop® system. For Asp biodegradation in August 2023, we note that the lower biodegradation extent was ascribed to an early onset of biodegradation (during setting up the experiment). **(B)** and **(C)** Times to reach 10% and 50% biodegradation, respectively. The data for March and August 2023 is identical to the standard experimental data depicted in **Figure 1** and **3**, respectively. Gluc: glucose, PEG: poly(ethylene glycol), Lys: lysine, PLL: ε-poly(L-lysine), Asp: aspartic acid, PAsA: poly(aspartic acid), PVA: poly(vinyl alcohol).

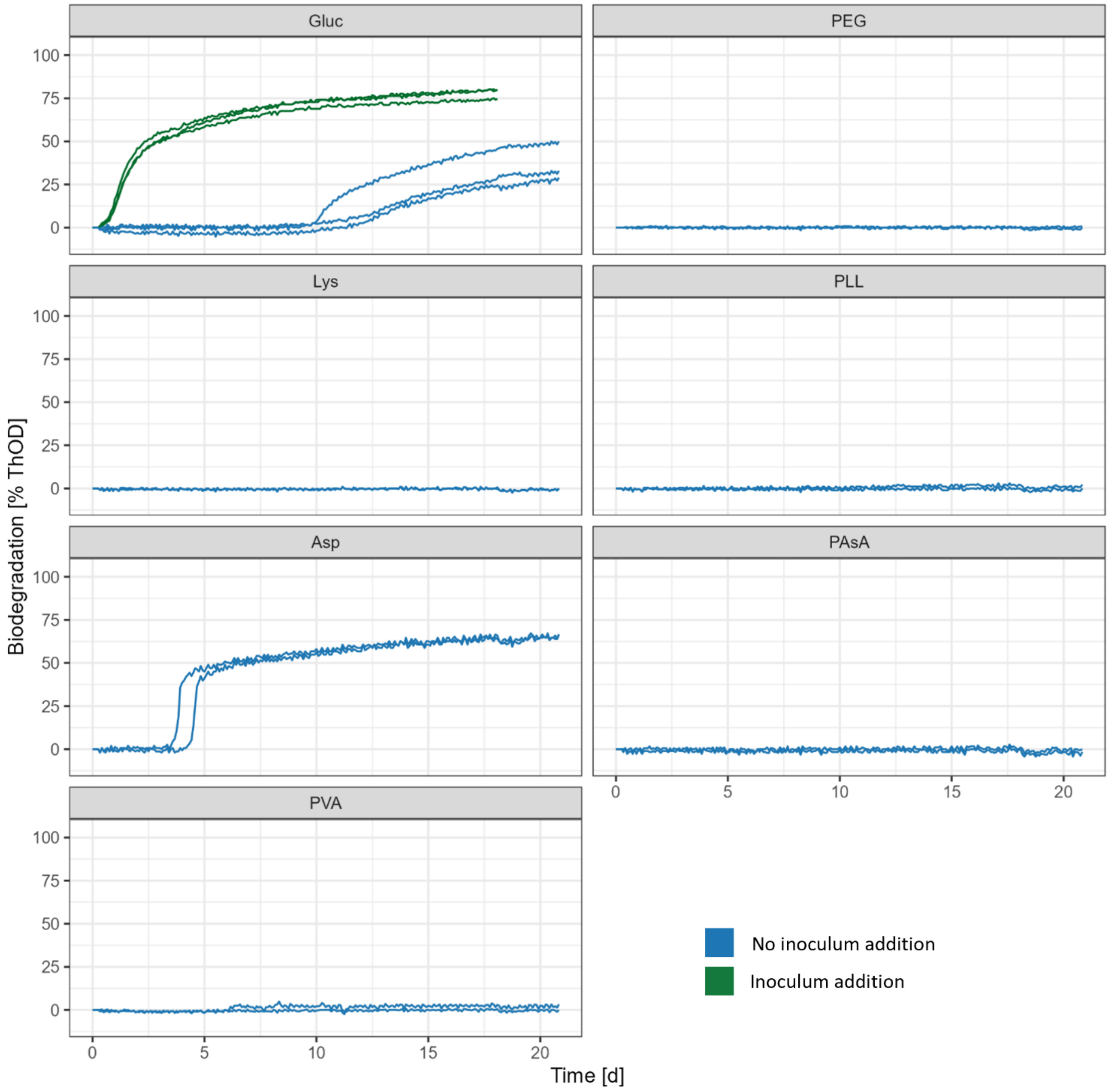

Figure S9. Abiotic control experiment for WSPs and low-molecular weight reference compounds. Mineralization curves calculated based on theoretical O_2 demand (ThOD) and measured O_2 consumption during WSP incubation using the OxiTop® system. A positive control with microbial inoculum from wastewater treatment plant (WWTP) 1 was run for glucose (shown in green). Gluc: glucose, PEG: poly(ethylene glycol), PVA: poly(vinyl alcohol), Lys: lysine, PLL: ε-poly(L-lysine), Asp: aspartic acid, PAsA: poly(aspartic acid).

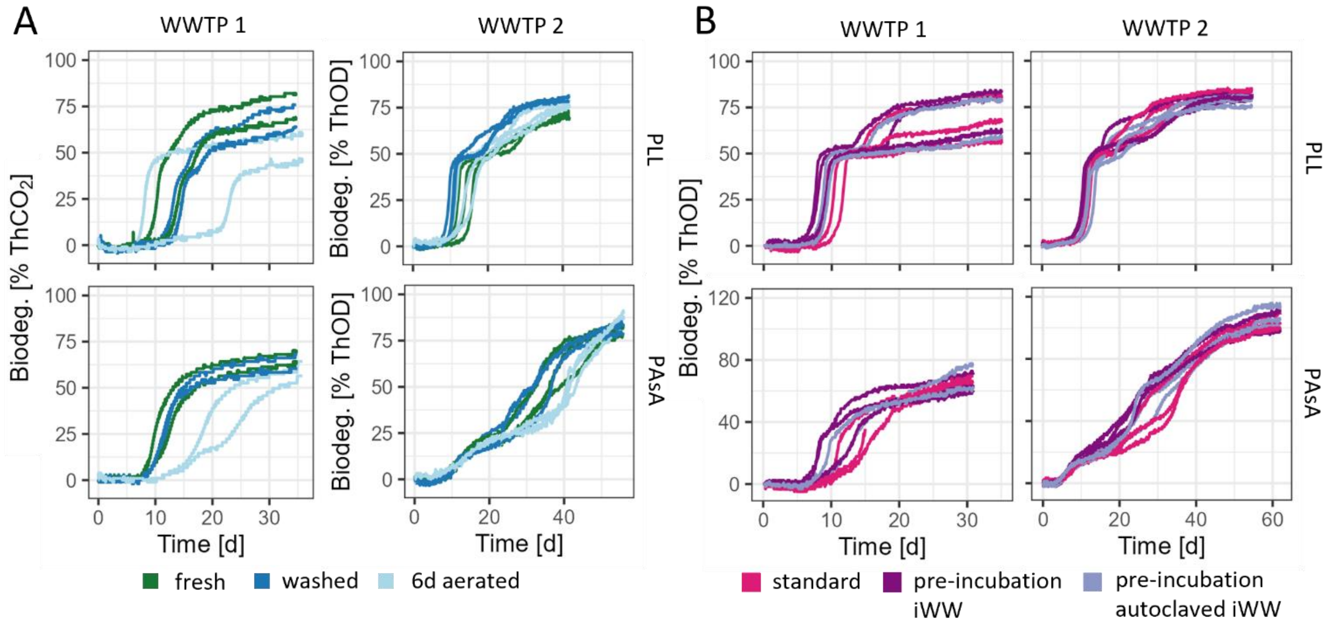

Figure S10. Effect of different protocol adaptations on poly(amino acids) biodegradation. **(A)** Effect of inoculum washing and aeration (for 6 days) on WSP biodegradation. Biodegradation curves were calculated based on theoretical CO_2 production ($ThCO_2$) using the BSBdigi-CO_2® system for wastewater treatment plant (WWTP) 1 inocula and based on theoretical O_2 demand (ThOD) using the OxiTop® system for WWTP 2 inocula. **(B)** Effect of pre-incubation with filter-sterilized influent wastewater (iWW) on WSP biodegradation. Biodegradation curves calculated based on theoretical O_2 demand (ThOD) and measured O_2 consumption using the OxiTop® system for WWTP 1 and 2 inocula. Standard curves for WWTP 2 are based on the same data as shown in **Figure 1**. PLL: ε-poly(L-lysine). PAsA: poly(aspartic acid).

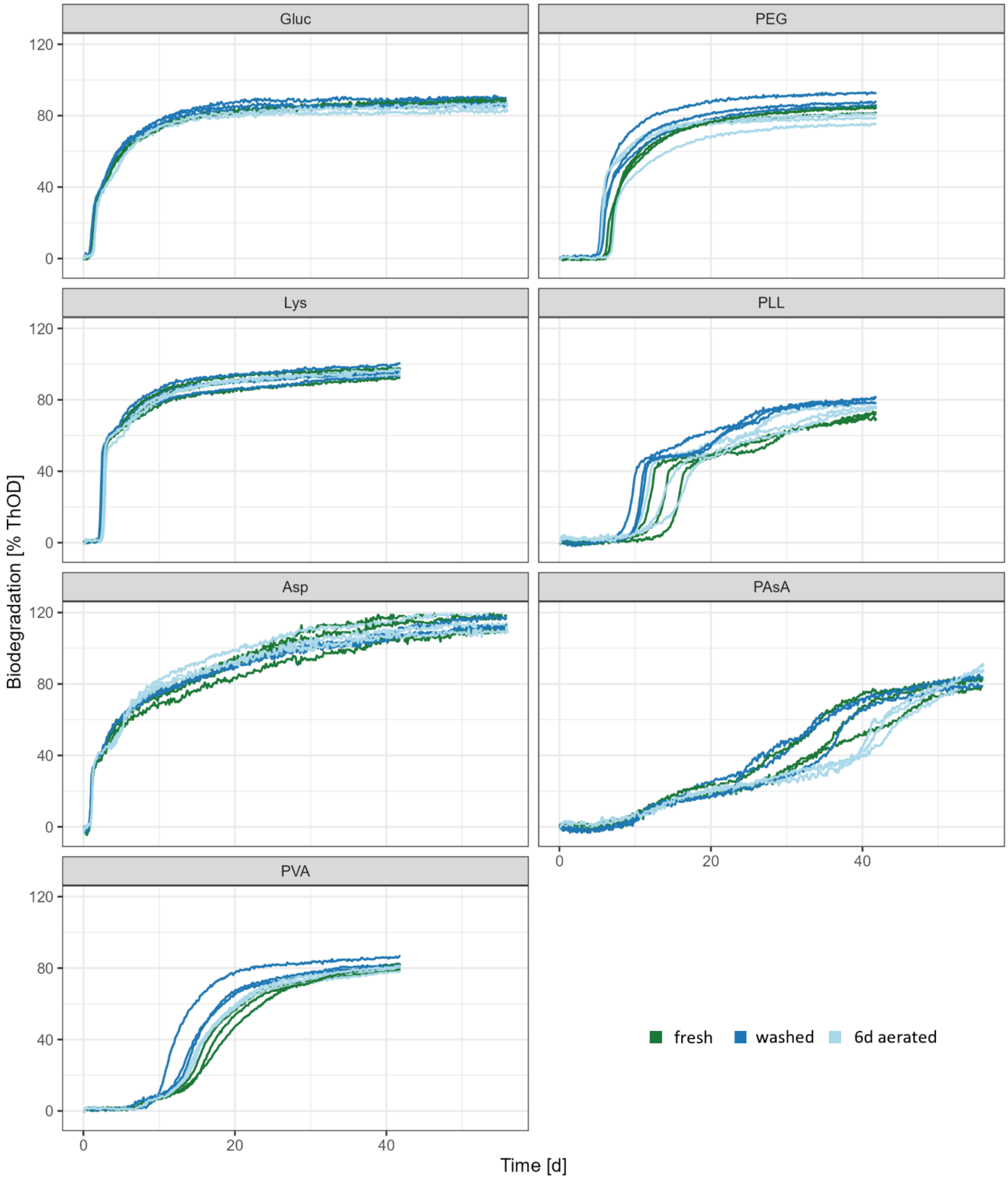

Figure S11. Effect of inoculum washing and aeration (6 days) on WSP biodegradation using inoculum from wastewater treatment plant (WWTP) 2. Biodegradation curves calculated based on theoretical O_2 demand (ThOD) and measured O_2 consumption during WSP incubation using the OxiTop® system. All measurements were conducted in triplicates (n=3). The data for PLL and PAsA is the same as shown in Figure 2. Gluc: glucose, PEG: poly(ethylene glycol), PVA: poly(vinyl alcohol), Lys: lysine, PLL: ε-poly(L-lysine), Asp: aspartic acid, PAsA: poly(aspartic acid).

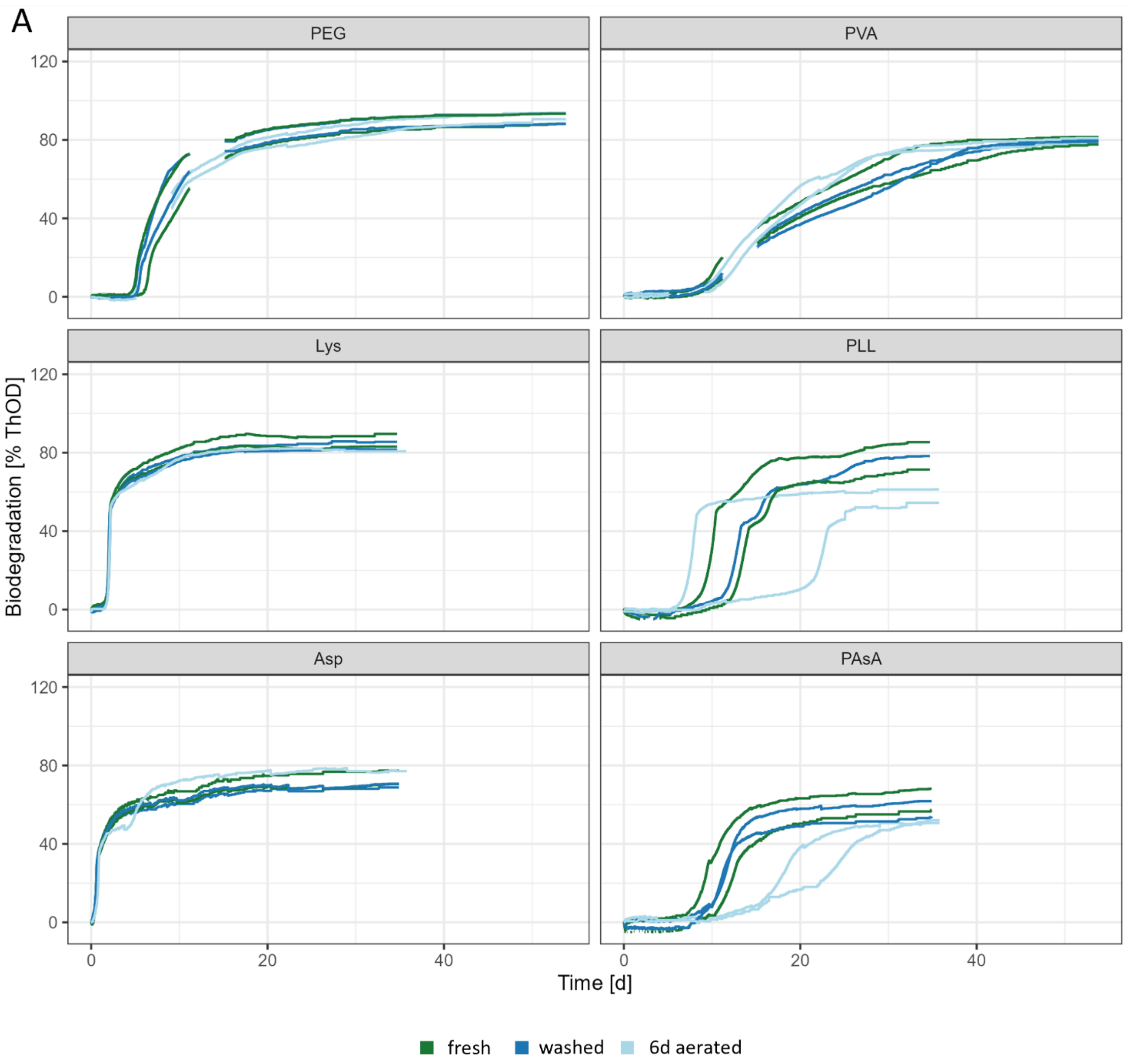

Figure S12; continued on next page

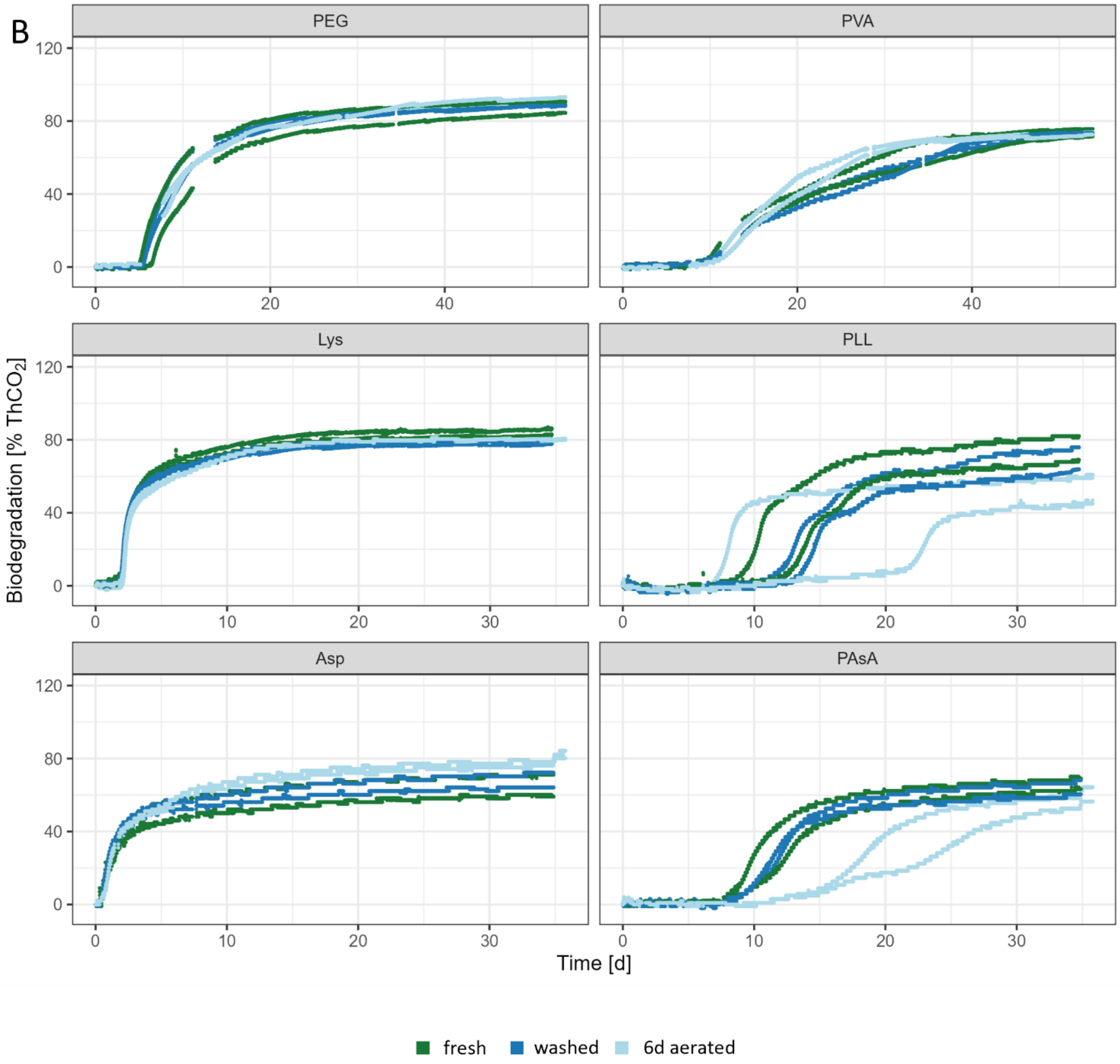

Figure S12. Effect of inoculum washing and aeration (6 days) on WSP biodegradation using inoculum from wastewater treatment plant (WWTP) 1. **(A)** Biodegradation curves calculated based on measured (with BSBdigi-CO_2®) and theoretical O_2 demand (ThOD). **(B)** Biodegradation curves calculated based on measured (with BSBdigi-CO_2®) and theoretical CO_2 production ($ThCO_2$). All measurements were conducted in duplicates (n=2). The data was collected during three separate experiments (i.e., one experiment for PEG and PVA, one experiment for lysine and PLL, one experiment for aspartic acid and PAsA). The data for PLL and PAsA is the same as shown in Figure 2. Gluc: glucose, PEG: poly(ethylene glycol), PVA: poly(vinyl alcohol), Lys: lysine, PLL: ε-poly(L-lysine), Asp: aspartic acid, PAsA: poly(aspartic acid)..

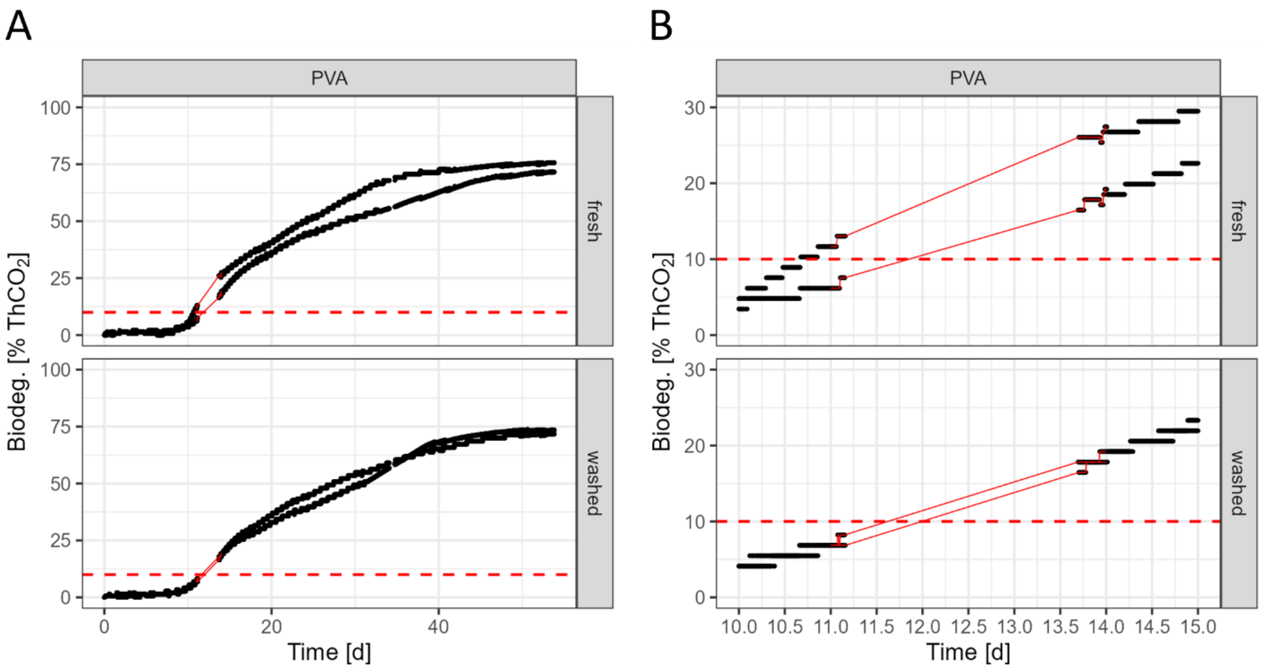

Figure S13. Linear interpolation for data gaps caused by BSBdigi-CO_2® software malfunctioning. Experiment: Effect of inoculum washing and aeration (6 days) on PVA biodegradation. **(A)** Biodegradation curves calculated based on measured (with BSBdigi-CO_2®) and theoretical CO_2 production ($ThCO_2$) for wastewater treatment plant (WWTP) 1. **(B)** Close-up. The dashed red line signifies the 10% biodegradation threshold, while the continuous red lines represent linear interpolation between data points. PVA: poly(vinyl alcohol).

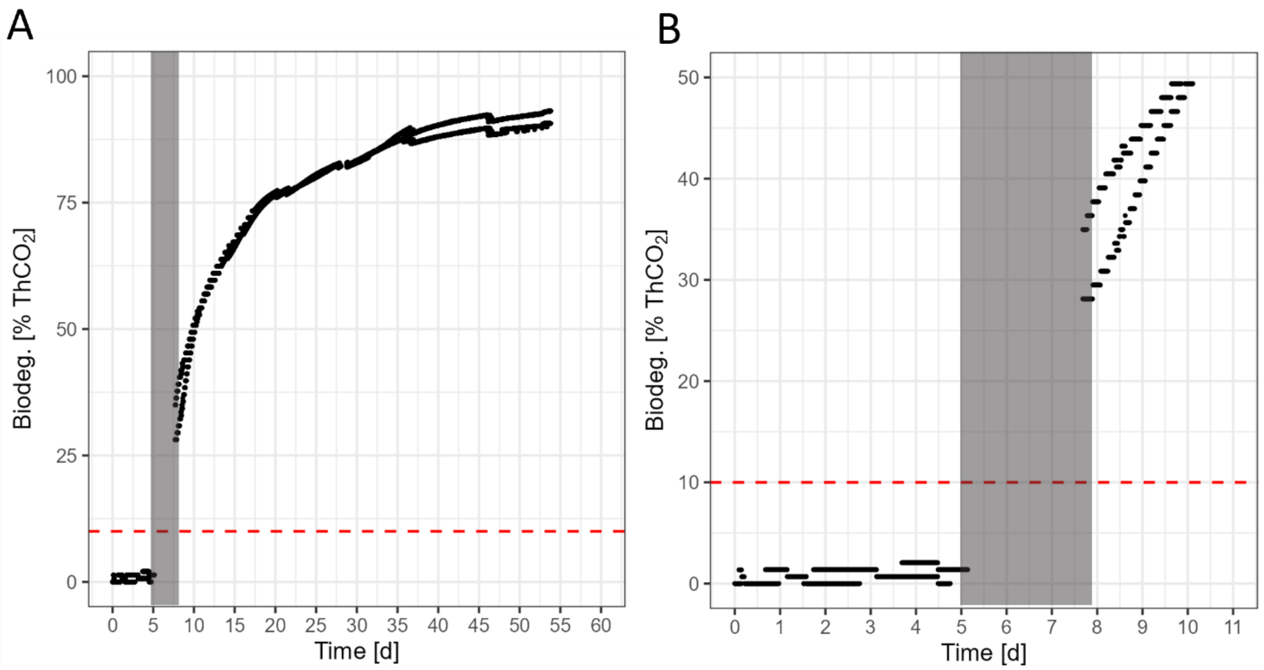

Figure S14. Visual determination of the time to reach 10% biodegradation for PEG using 6-day aerated inoculum. The data gap occurred due to BSBdigi-CO2® software malfunction. The dashed red line represents the 10% biodegradation threshold. (**A**) Biodegradation curves based on measured (with BSBdigi-CO_2®) and theoretical CO_2 production ($ThCO_2$) for wastewater treatment plant (WWTP) 1. (**B**) Close-up view. Visual examination indicates that 10% biodegradation was reached between Day 5 and Day 7.5. PEG: poly(ethylene glycol).

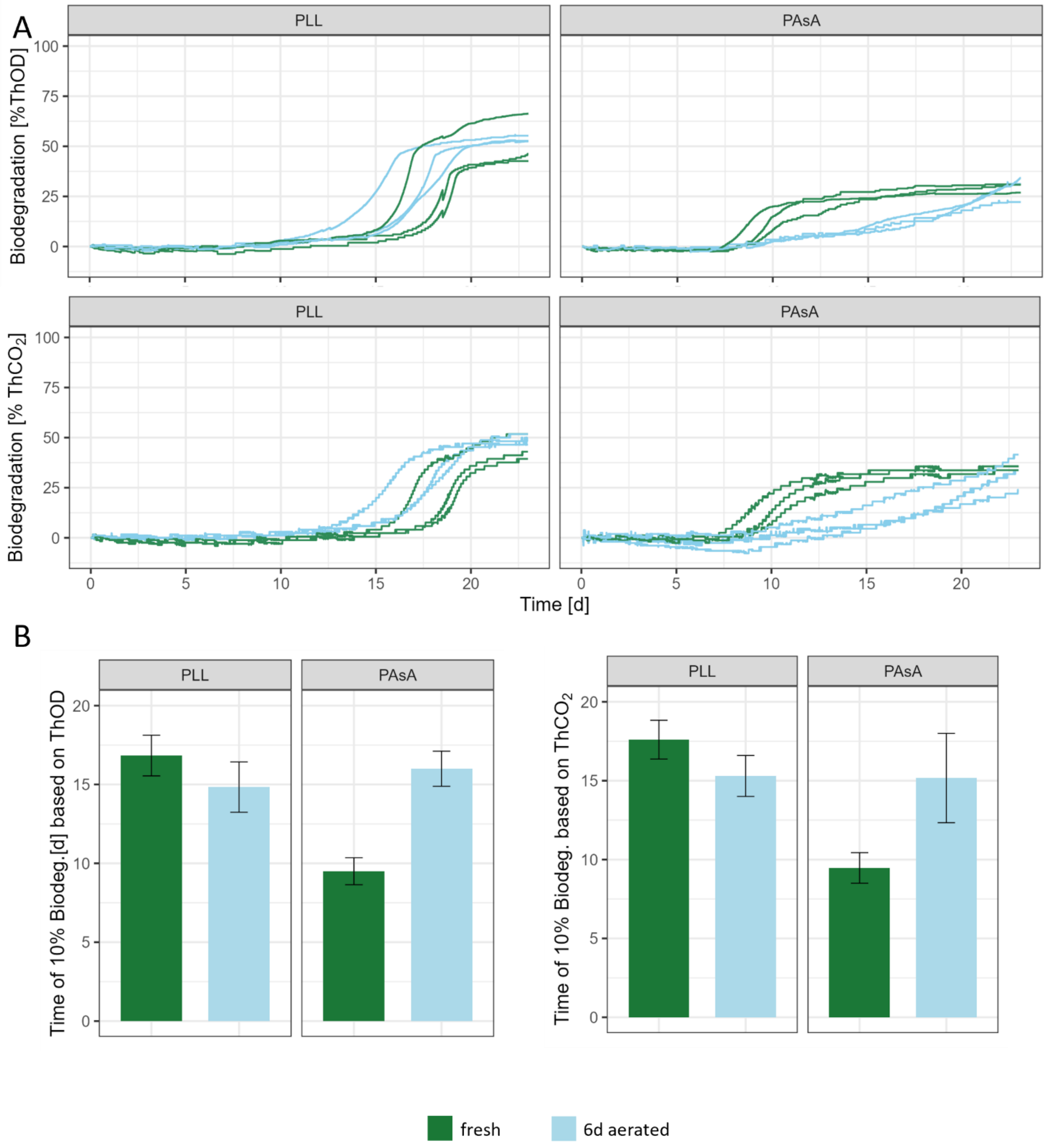

Figure S15. Repetition: Comparison of PAsA and PLL biodegradation with fresh and 6-day aerated inoculum from wastewater treatment plant (WWTP) 1. **(A)** Biodegradation curves calculated based on measured (with BSBdigi-CO_2®) and theoretical O_2 demand (ThOD) and theoretical CO_2 production ($ThCO_2$). **(B)** Times to reach 10% biodegradation. A two-sided t-test comparing fresh and aerated inoculum revealed a significant difference for PAsA ($p = 0.0017$) based on ThOD measurements. No significant difference was observed based on CO_2 measurements ($p = 0.06$). No significant difference was observed between fresh and aerated sludge for PLL. PLL: ε-poly(L-lysine), PAsA: poly(aspartic acid).

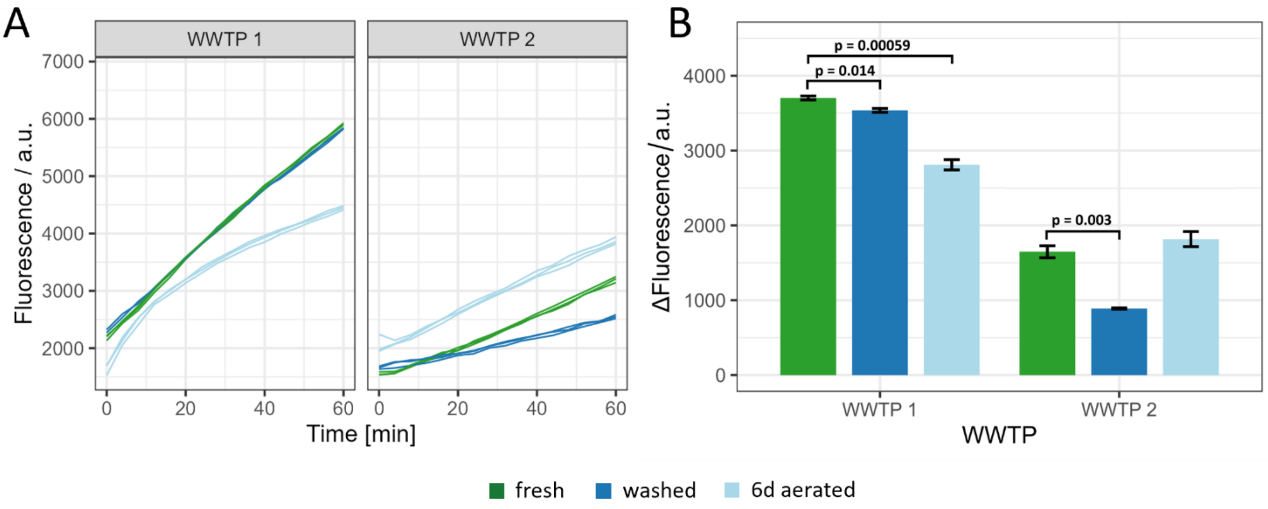

Figure S16. Effect of inoculum washing and aeration (6 days) on peptidase activity measured with EnzChek Protease Assay Kit (Thermo Fisher, E6638). Microbial inocula were obtained from the aeration tank of wastewater treatment plant (WWTP) 1 and 2. (**A**) Fluorescence intensity over time. (**B**) Change (Δ) in fluorescence intensity between 0 and 60 minutes of incubation. Bars and error bars represent means and standard deviations of triplicate assay measurements. The lines above the bars denote the results of a two-sided t-test, highlighting statistically significant differences in fluorescence intensity between washed or 6d aerated sludge relative to the fresh sludge ($p < 0.05$).

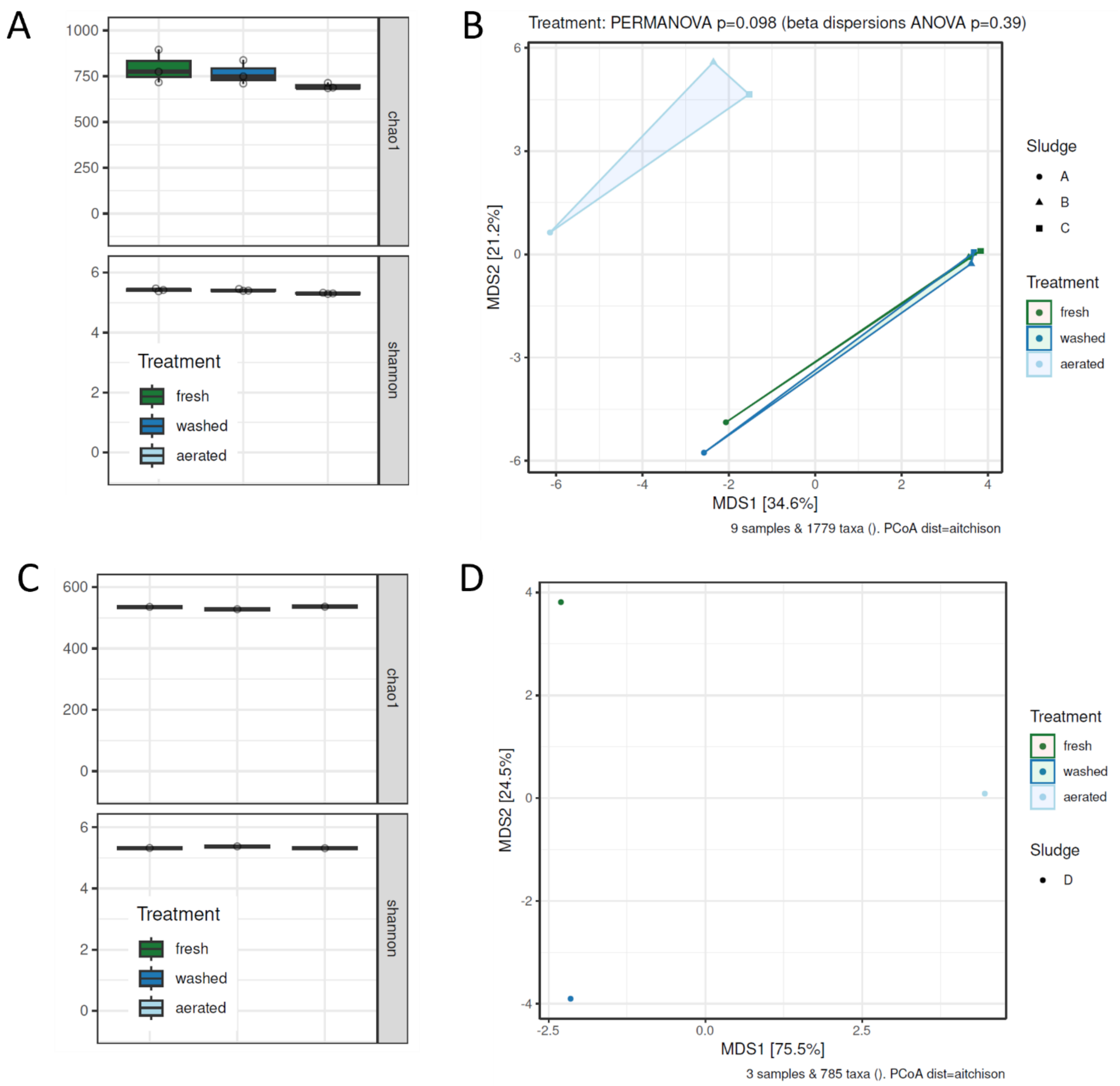

Figure S17. 16S rRNA community analysis for sludge samples from wastewater treatment plant (WWTP) 1 and 2. (**A**) WWTP1: Alpha and beta-diversity. (**B**) WWTP1: Multidimensional scaling (MDS) to visualize the differences in community composition between samples. (**C**) WWTP2: Alpha and beta-diversity. (**D**) WWTP2: Multidimensional scaling (MDS) to visualize the differences in community composition between samples.

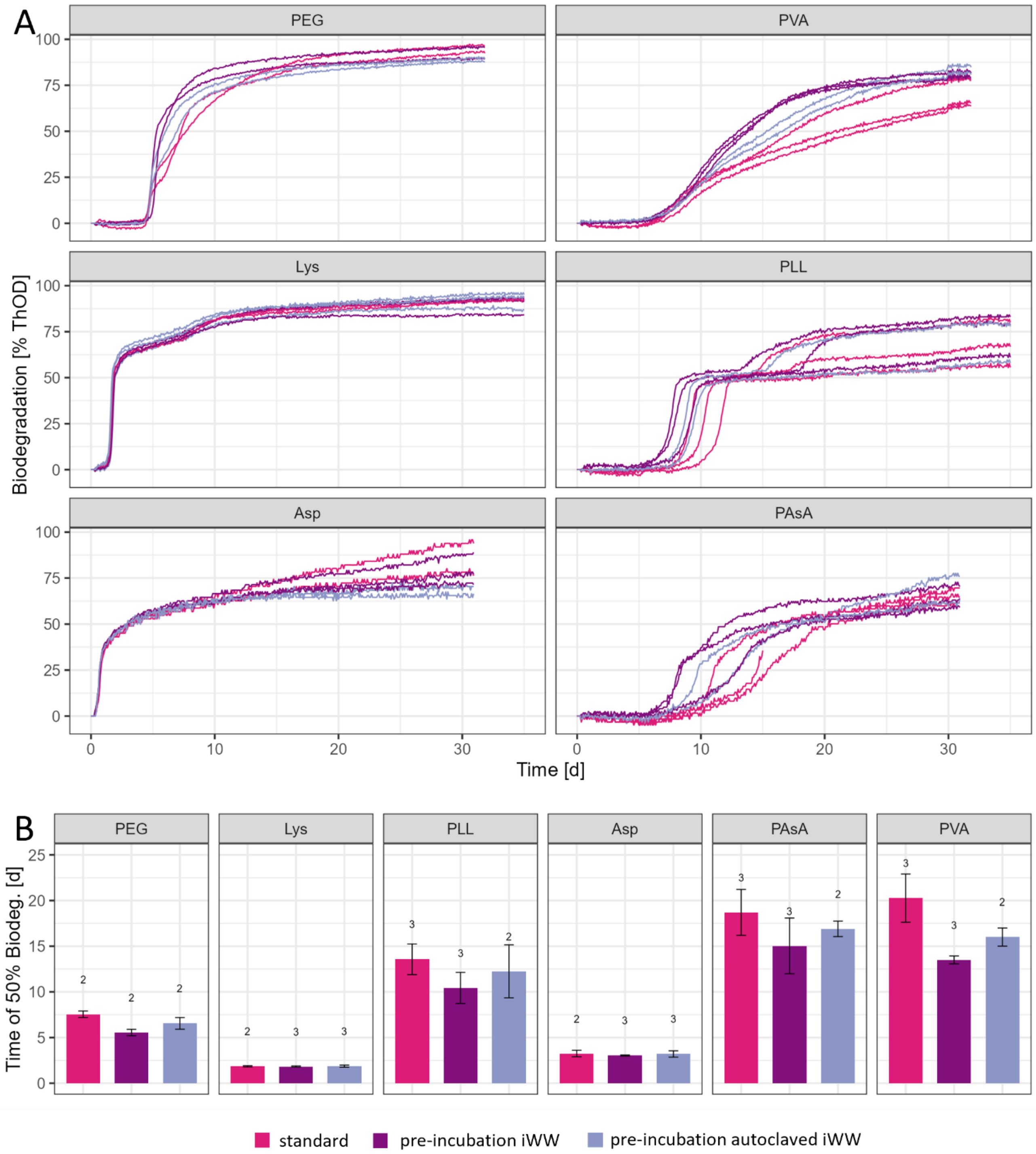

Figure S18. Effect of pre-incubation with filter-sterilized influent wastewater (iWW) on WSP biodegradation using inoculum from wastewater treatment plant (WWTP) 1. **(A)** Biodegradation curves calculated based on theoretical O_2 demand (ThOD) and measured O_2 consumption during WSP incubation using the OxiTop® system. Data for PLL and PAsA is the same as shown in **Figure 3**. **(B)** Times required to reach 50% biodegradation. Error bars represent standard deviations for triplicates and ranges for duplicates. The number of replicates (n= x) is provided above the bar. PEG: poly(ethylene glycol), PVA: poly(vinyl alcohol), Lys: lysine, PLL: ε-poly(L-lysine), Asp: aspartic acid, PAsA: poly(aspartic acid).

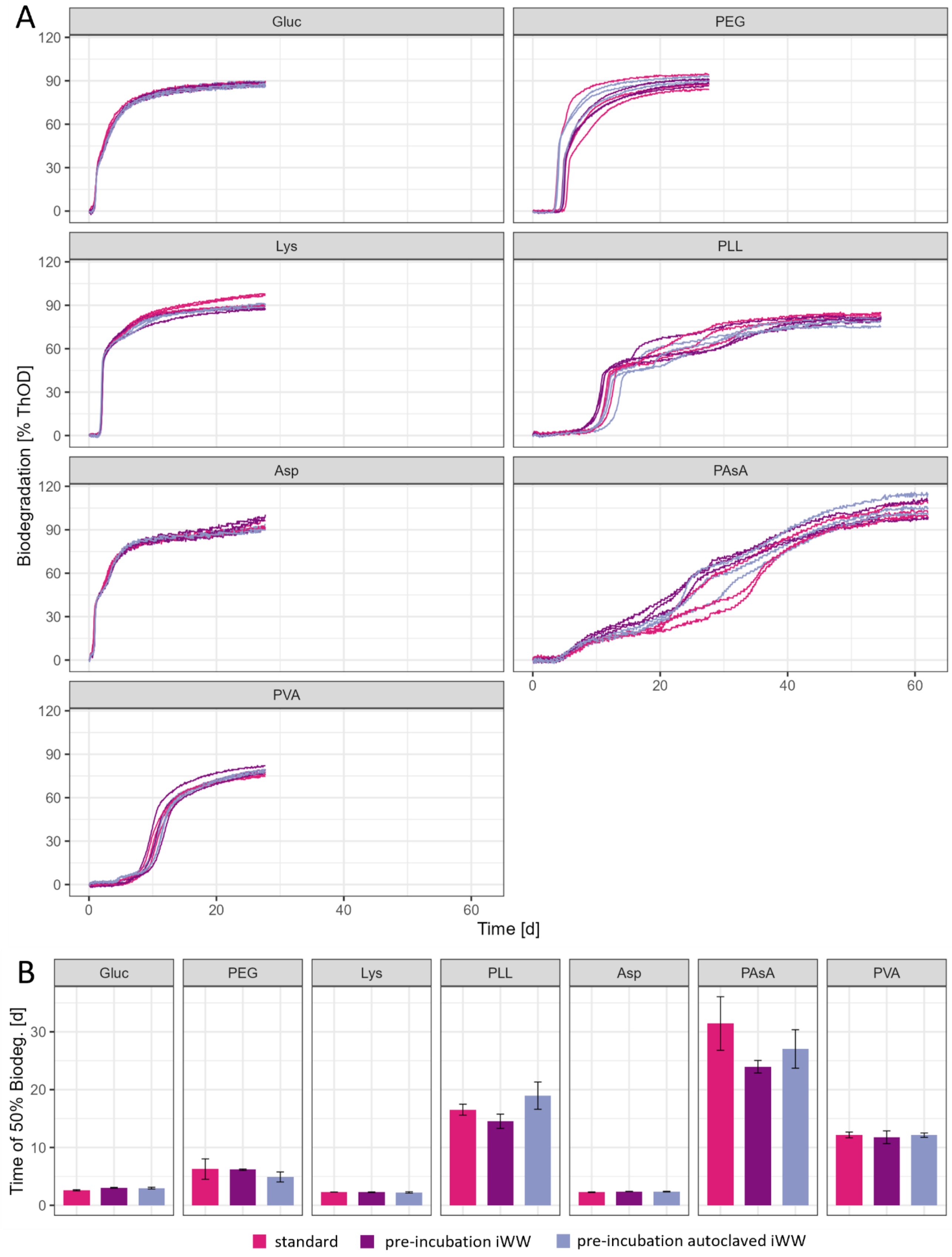

Figure S19. Effect of pre-incubation with filter-sterilized influent wastewater (iWW) on WSP biodegradation using inoculum from wastewater treatment plant (WWTP) 2. **(A)** Biodegradation curves calculated based on theoretical O_2 demand (ThOD) and measured O_2 consumption during WSP incubation

using the OxiTop® system. Data for PLL and PAsA is the same as shown in **Figure 3**. **(B)** Times required to reach 50% biodegradation. Error bars represent standard deviations of triplicates Gluc: glucose, PEG: poly(ethylene glycol), PVA: poly(vinyl alcohol), Lys: lysine, PLL: ε-poly(L-lysine), Asp: aspartic acid, PAsA: poly(aspartic acid).

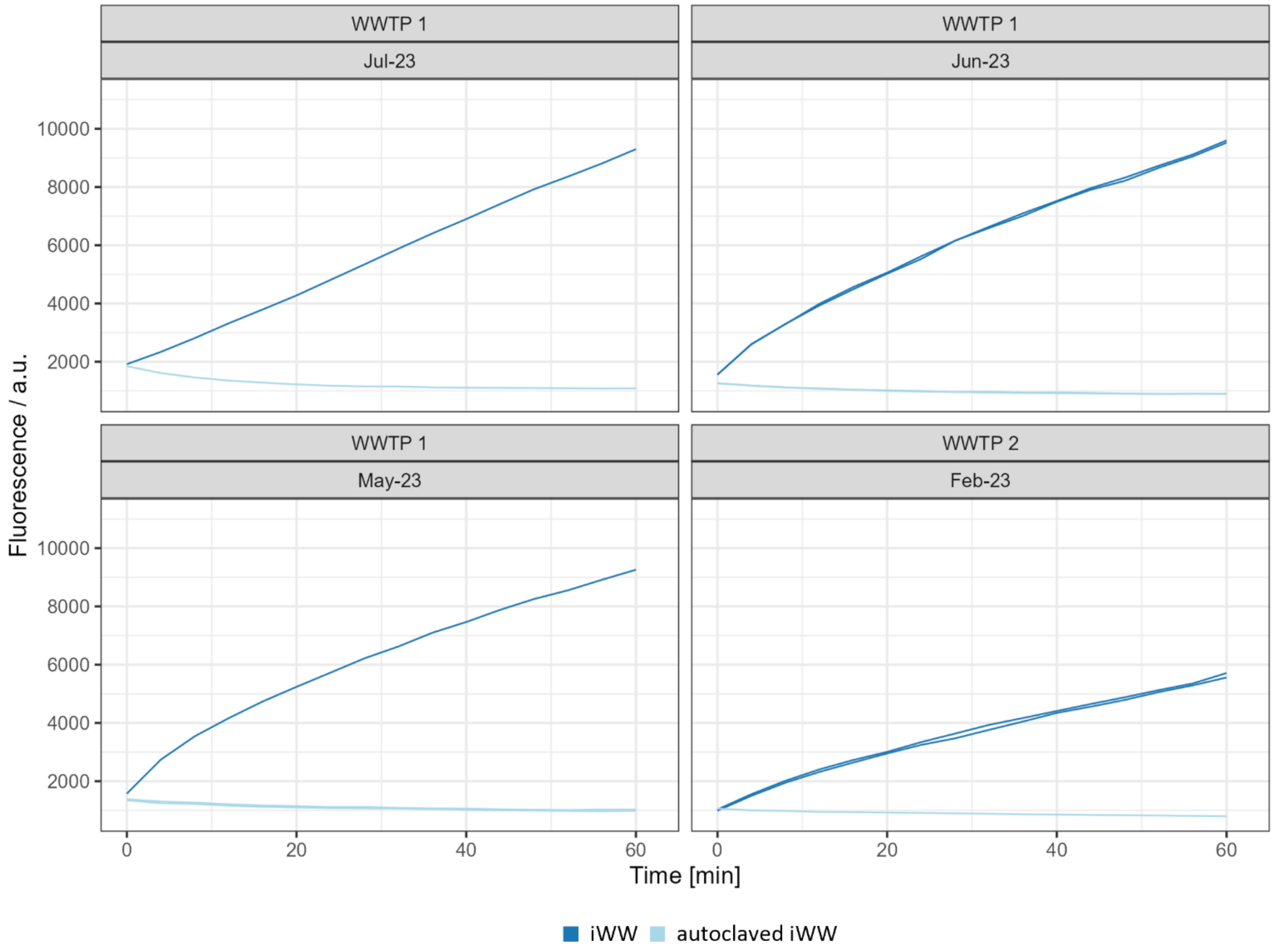

Figure S20. Peptidase activity of filter-sterilized influent wastewater (iWW) measured with EnzChek Protease Assay Kit (Thermo Fisher, E6638). The figure shows the fluorescence signal over time in active and autoclaved iWW samples wastewater treatment plant (WWTP) 1 and 2.

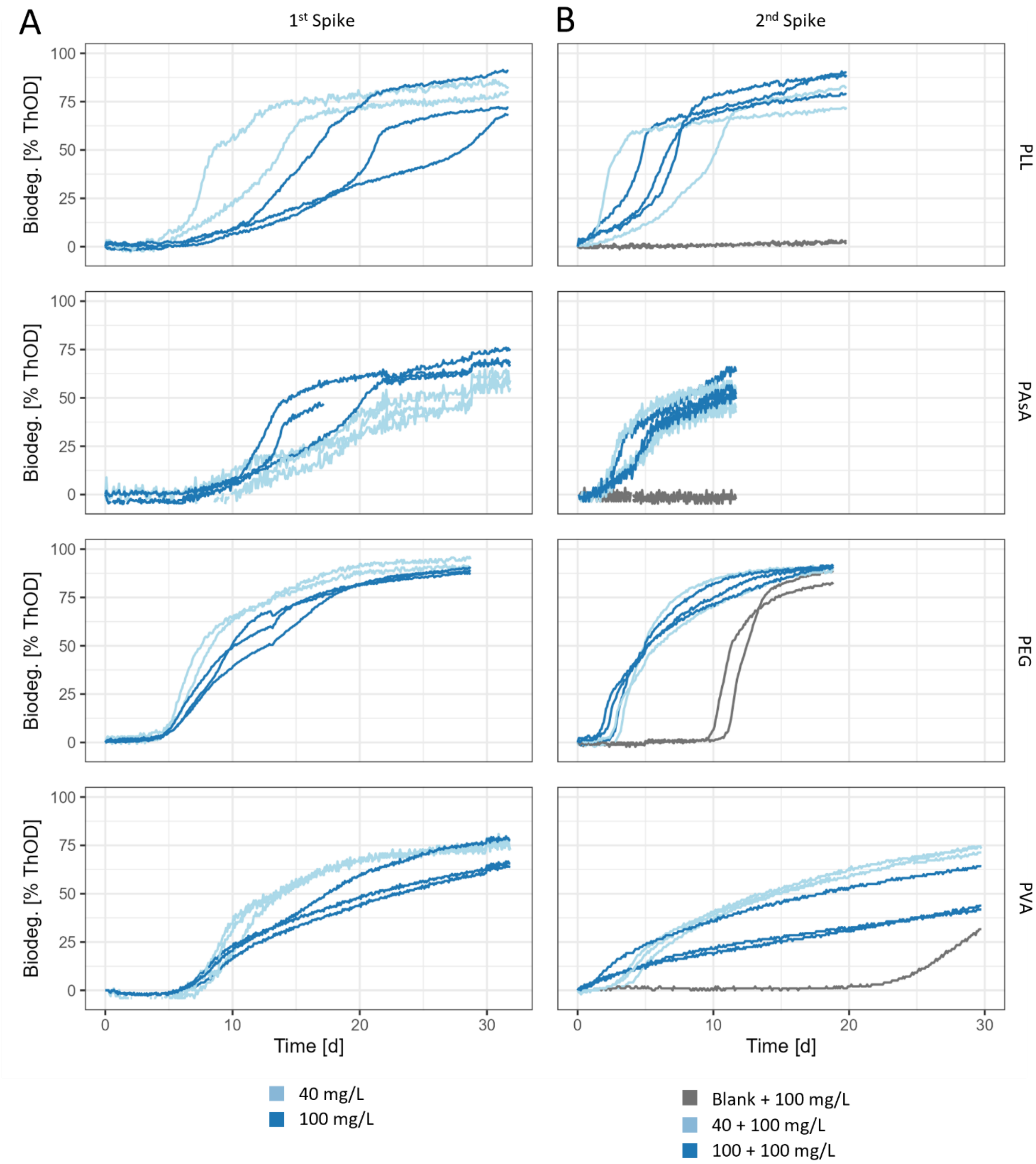

Figure S21. WSP biodegradation at different concentrations and pre-exposure using inoculum from wastewater treatment plant (WWTP) 1. (**A**) Biodegradation curves calculated based on theoretical O_2 demand (ThOD) and measured O_2 consumption during WSP incubation using the OxiTop® system for two concentrations: 40 and 100 mg polymer/L. (**B**) Biodegradation curves of the same experiments as shown in panel (A), where at the last time point in panel (A), which equals time = 0 days in panel (B), substrate (to a concentration of 100 mg/L) was added a second time. PLL: ε-poly(L-lysine), PAsA: poly(aspartic acid), PEG: poly(ethylene glycol), PVA: poly(vinyl alcohol).

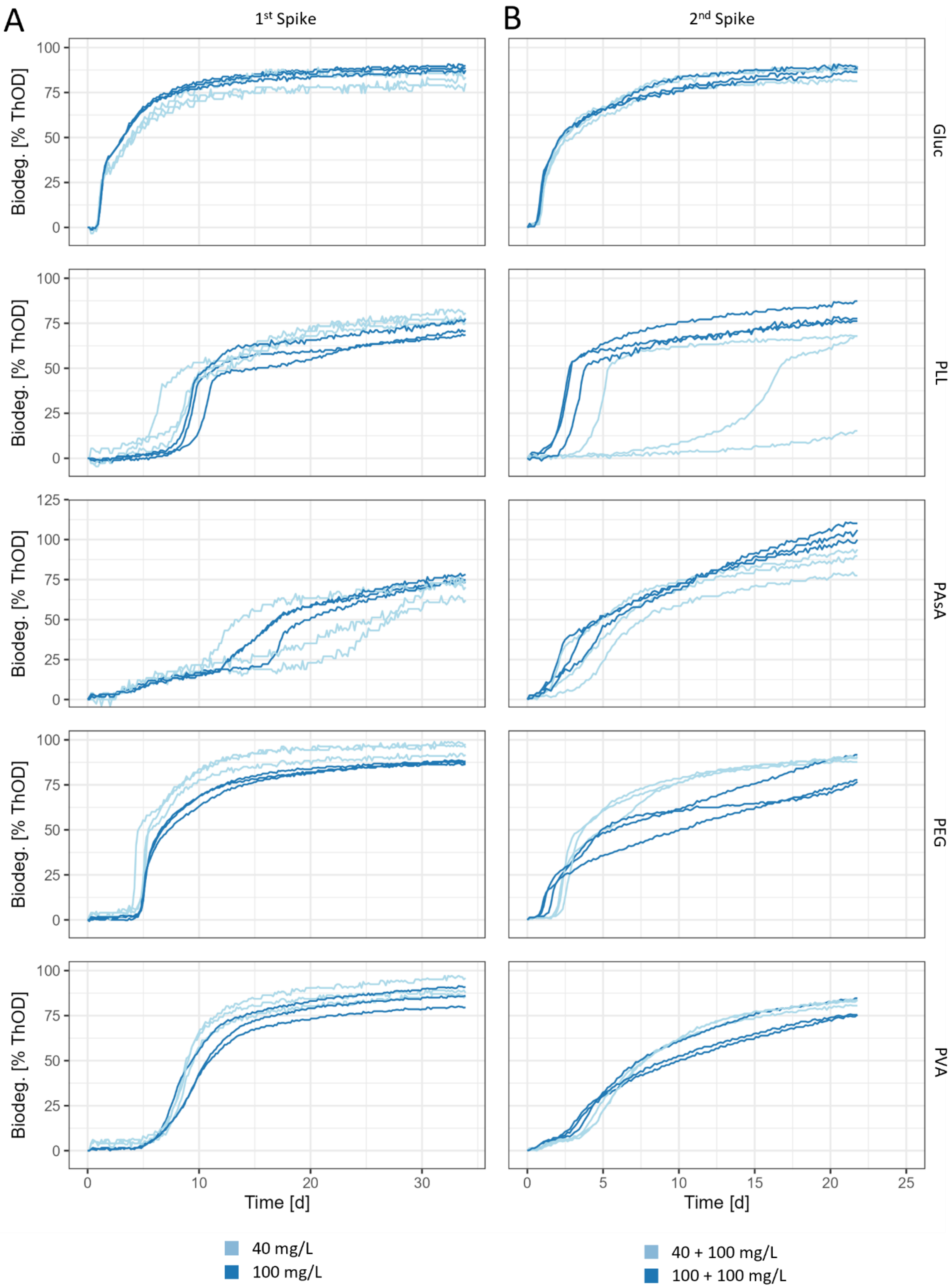

Figure S22. WSP biodegradation at different concentrations and pre-exposure using inoculum from wastewater treatment plant (WWTP) 2. (**A**) Biodegradation curves calculated based on theoretical O_2 demand (ThOD) and measured O_2 consumption during WSP incubation using the OxiTop® system for two concentrations: 40 and 100 mg substrate/L. (**B**) Biodegradation curves of the same experiments as shown

in panel (A), where at the last time point in panel (A), which equals time = 0 days in panel (B), substrate (to a concentration of 100 mg/L) was added a second time. Gluc: glucose, PLL: ε-poly(L-lysine), PAsA: poly(aspartic acid), PEG: poly(ethylene glycol), PVA: poly(vinyl alcohol)

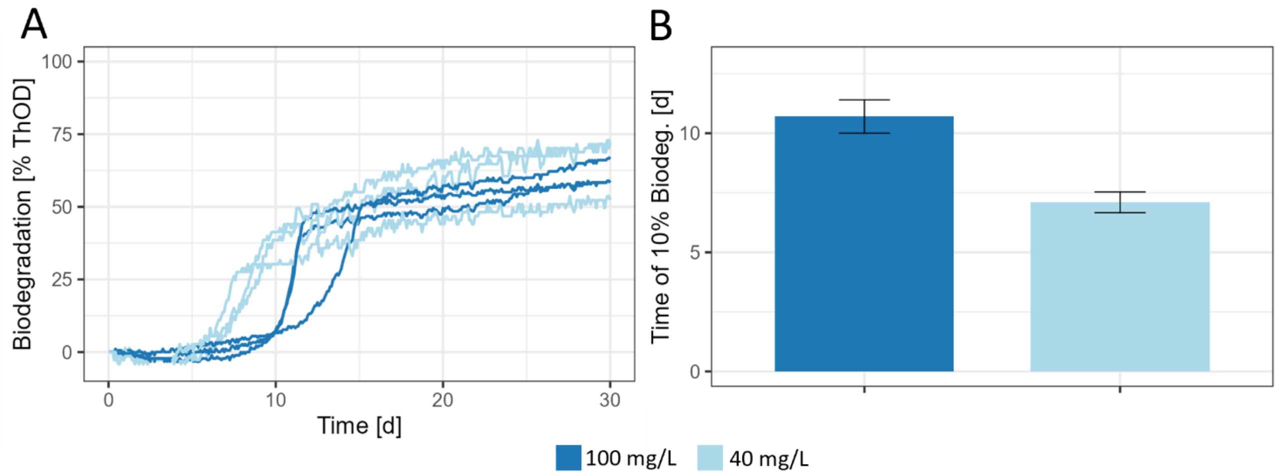

Figure S23. Repetition: PLL biodegradation at 40 and 100 mg/L with inoculum from wastewater treatment plant (WWTP) 1. (**A**) Biodegradation curves calculated based on theoretical O_2 demand (ThOD) and measured O_2 consumption during WSP incubation using the OxiTop® system. **(B)** Times required to reach 10% biodegradation. Error bars represent standard deviations of triplicates. A two-sided t-test comparing the lag phases revealed a significant difference between the two concentrations (p= 0.003). PLL: ε-poly(L-lysine).

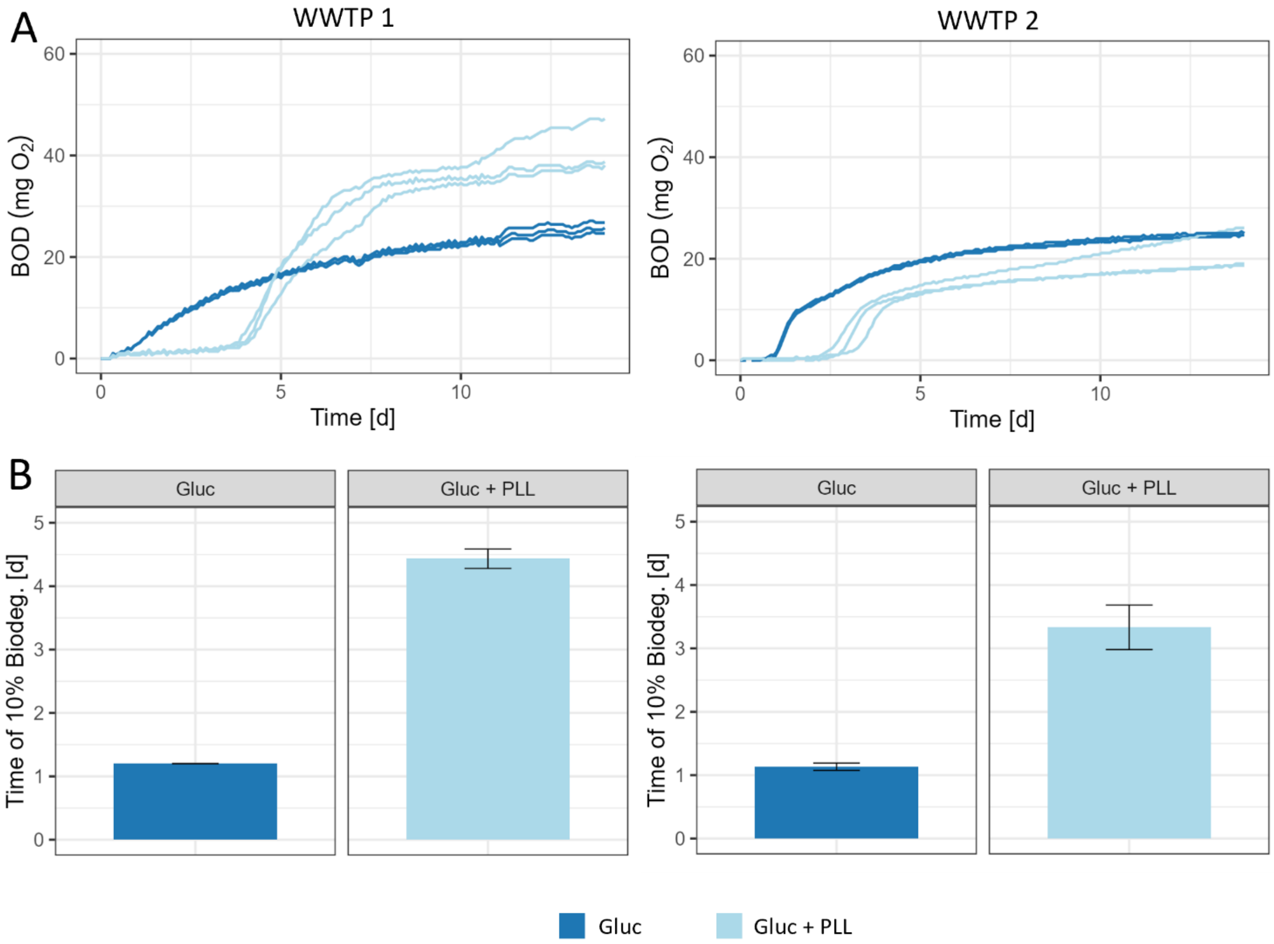

Figure S24. PLL inhibitory test with sludge from both wastewater treatment plants (WWTPs). **(A)** Biological oxygen demand (BOD) curves obtained from the OxiTop® system for glucose in the absence (Gluc) and presence of 100 mg/L PLL (Gluc + PLL). **(B)** Times required to reach 10% biodegradation. Error bars represent standard deviations of triplicates. A two-sided t-test comparing the lag-phase of Gluc and Gluc + PLL resulted in significant differences (WWTP 1: $p = 0.0007$; WWTP 2: $p = 0.007$). Gluc: glucose, PLL: ε-poly(L-lysine).

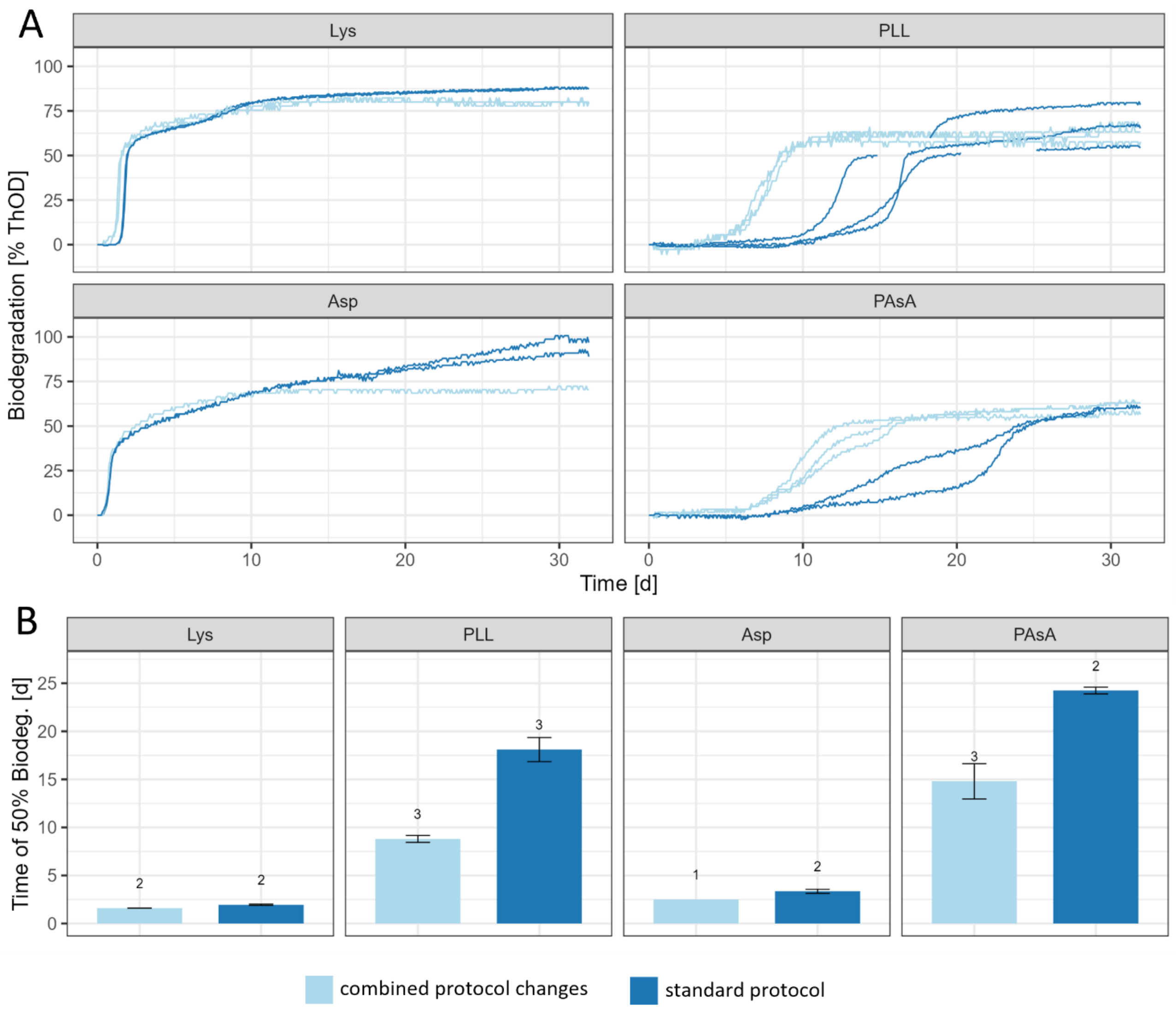

Figure S25. Combined effects of sludge aeration, pre-incubation with filter-sterilized influent wastewater (iWW), and concentration on biodegradation using inoculum from wastewater treatment plant (WWTP) 1. **(A)** Biodegradation curves calculated based on theoretical O_2 demand (ThOD) and measured O_2 consumption during WSP incubation using the OxiTop® system. **(B)** Times required to reach 50% biodegradation. Error bars represent standard deviations of replicates (n = x, indicated above each bar). The data for PLL and PAsA is the same as shown in **Figure 4**. Lys: lysine, PLL: ε-poly(L-lysine), Asp: aspartic acid, PAsA: poly(aspartic acid).

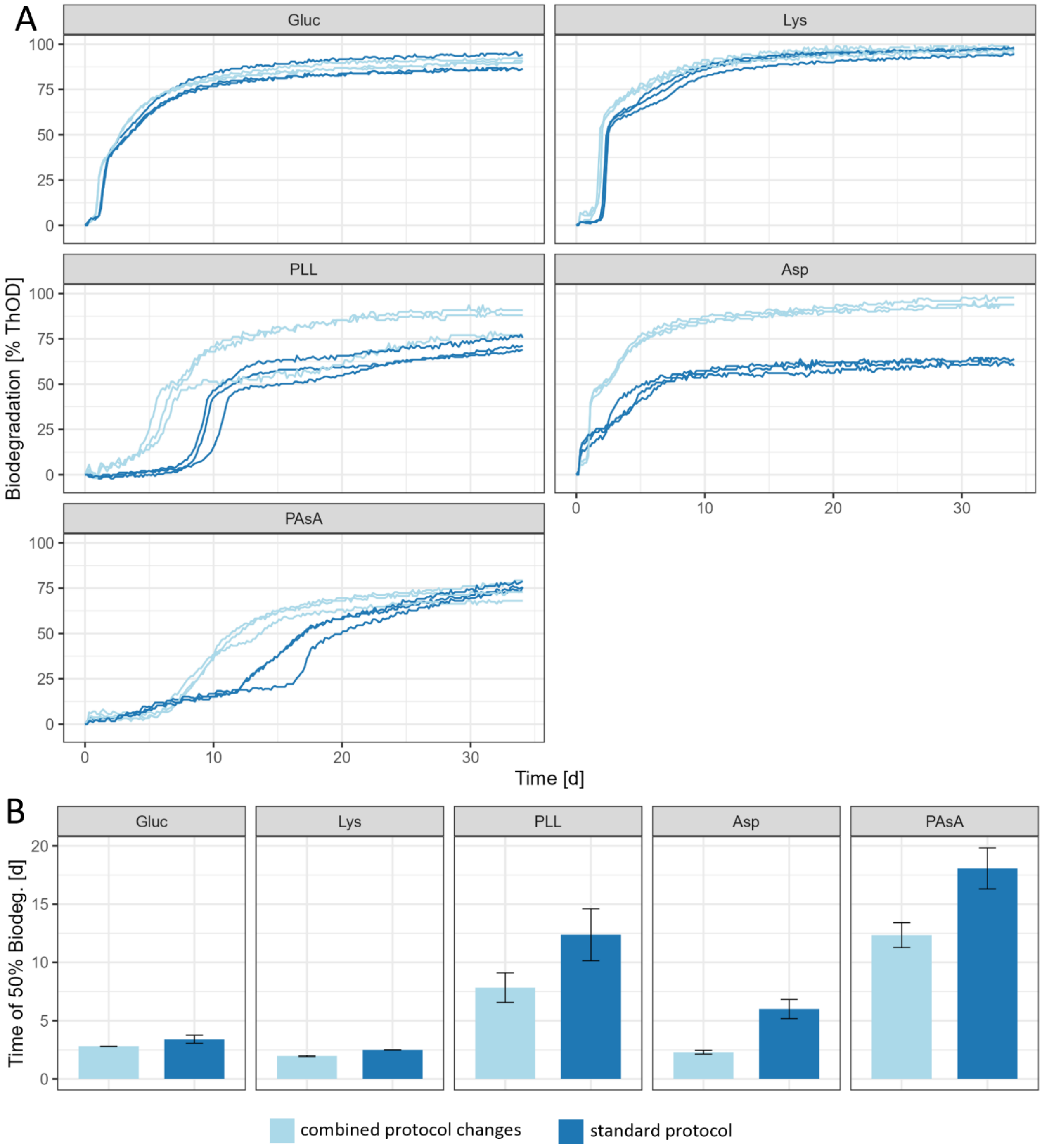

Figure S26. Combined effects of sludge aeration, pre-incubation with filter-sterilized influent wastewater (iWW), and concentration on biodegradation using inoculum from wastewater treatment plant (WWTP) 2. **(A)** Biodegradation curves calculated based on theoretical O_2 demand (ThOD) and measured O_2 consumption during WSP incubation using the OxiTop® system. For Asp biodegradation in August 2023, we note that the lower biodegradation extent was ascribed to an early onset of biodegradation (during setting up the experiment). **(B)** Times required to reach 10% biodegradation. Error bars represent standard deviations of triplicates. The data for PLL and PAsA is the same as shown in **Figure 4**. Gluc: glucose, Lys: lysine, PLL: ε-poly(L-lysine), Asp: aspartic acid, PAsA: poly(aspartic acid).

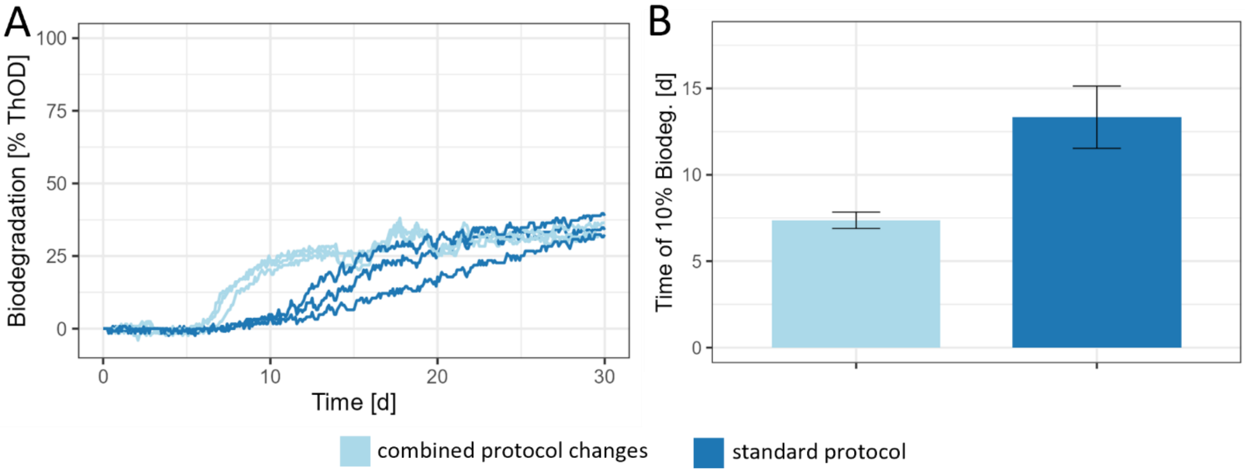

Figure S27. Repetition: PAsA biodegradation with standard protocol and combined protocol changes conditions for wastewater treatment plant (WWTP) 1. **(A)** Biodegradation curves calculated based on measured (with BSBdigi-CO_2®) and theoretical O_2 demand (ThOD) and theoretical CO_2 production ($ThCO_2$). **(B)** Times to reach 10% biodegradation. A two-sided t-test comparing the standard protocol with the combined protocol changes revealed a significant difference for PAsA ($p= 0.023$). PAsA: poly(aspartic acid).

**Substrate**	**Abb.**	**Formula**	**Average MW [kDa]**	**Source (article number)**	**Solid content (%)**	**Elemental analysis**								
						C	H	O	Cl	N	Na	S	P	Sum EA [g/100g]
Polyethyleneglycol	PEG	$H(OCH_2CH_2)_nOH$	6	BASF	100	52.6	9.4	37.7						99.70
Polyvinyl alcohol (87-90% hydrolyzed)	PVA	$(C_2H_4O)_n$	31-50	Sigma (363073)	100	52.5	9.1	38.2	0	0				99.80
epsilon-Poly(L-lysine) hydrochloride	PLL	$(C_6H_{12}N_2O{\cdot}HCl)_n$	3.5-4.5	Carbosynth (FP14985)	100	40.8	8.3	15.5	19.5	15.8				99.90
catalytic Poly(L-aspartic acid) sodium salt	PAsA	$(C_4H_5NO_3{\cdot}Na)_n$	13	BASF	40.22	14.2	7.9	62		4.4	6.7			95.20
						Theoretical elemental composition								
						C	H	O	Cl	N	Na	S	P	M [g/mol]
D-Glucose	Gluc	$C_6H_{12}O_6$		Sigma (G8270)	100	6	12	6						180
L-Lysine	Lys	$C_6H_{14}N_2O_2$		Sigma (L5501)	100	6	14	2		2				146
L-Aspartic acid	Asp	$C_4H_7NO_4$		Sigma (A9256)	100	4	7	4		1				133

Table S1. Substrate characteristics.

WWTP	WWTP1	WWTP 2
Location	Austria	Germany
Type	Activated sludge	Activated sludge
Resident values (EW)	55.000	725.000
Average wastewater quantity (daily load) [m^3]		90.000
Solid retention time A.T. 1 [d]	0.6	
Solid retention time A.T. 2 [d]	6.2	-
Hydraulic retention time A.T.1 [h]	2.2	22.1
Hydraulic retention time A.T.2 [h]	7.2	-

Table S2. WWTP characteristics.

WWTP	System	Sludge inoculum treatment	Replicate	Experiment duration [d]	Cumulative O_2 consumption [mg]	Cumulative CO_2 production [mg]
WWTP 1	BSBdigi-CO2	fresh	1	34.7	5.8	8.6
			2		5.1	7.9
		washed	1		4.5	7.9
			2		5.3	7.9
		aerated (6 days)	1		2.6	6.6
			2		4.0	5.9
WWTP1	BSBdigi-CO2	fresh	1	34.9	NA	10.6
			2		7.5	9.9
		washed	1		6.9	10.6
			2		8.6	9.3
		aerated (6 days)	1		3.7	5.9
			2		3.9	5.9
WWTP1	BSBdigi-CO2	fresh	1	53.8	3.4	7.9
			2		NA	8.6
		washed	1		3.1	7.9
			2		3.5	6.6
		aerated (6 days)	1		2.5	5.9
			2		2.5	6.6
WWTP 2	OxiTop	fresh	1	55.7	6.7	
			2		6.3	
			3		6.7	
		washed	1		6.3	
			2		5.6	
			3		6.7	
		aerated (6 days)	1		4.9	
			2		4.9	
			3		4.6	

Table S3. Effect of inoculum washing and aeration on blank background respiration. For WWTP 1 O_2 consumption and CO_2 production were assessed using the BSBdigi-CO_2® system, while for WWTP 2 the O_2 consumption was assessed with the OxiTop® system. Blank samples shown here belong to the experiments shown in **Figure 2A** and **Figure S11** and **S12**.

References SI

(1) OECD. *Test No. 301: Ready Biodegradability*; 1992. https://doi.org/10.1787/9789264070349-en.
(2) Parada, A. E.; Needham, D. M.; Fuhrman, J. A. Every Base Matters: Assessing Small Subunit RRNA Primers for Marine Microbiomes with Mock Communities, Time Series and Global Field Samples. *Environmental microbiology* **2016**, *18* (5), 1403–1414.
(3) Apprill, A.; McNally, S.; Parsons, R.; Weber, L. Minor Revision to V4 Region SSU RRNA 806R Gene Primer Greatly Increases Detection of SAR11 Bacterioplankton. *Aquatic Microbial Ecology* **2015**, *75* (2), 129–137.
(4) Pjevac, P.; Hausmann, B.; Schwarz, J.; Kohl, G.; Herbold, C. W.; Loy, A.; Berry, D. An Economical and Flexible Dual Barcoding, Two-Step PCR Approach for Highly Multiplexed Amplicon Sequencing. *Front. Microbiol.* **2021**, *12*, 669776. https://doi.org/10.3389/fmicb.2021.669776.
(5) Demultiplex: FASTA/FASTQ Demultiplexer. https://github.com/jfjlaros/demultiplex (accessed 2024-05-31).
(6) Callahan, B. J.; McMurdie, P. J.; Rosen, M. J.; Han, A. W.; Johnson, A. J. A.; Holmes, S. P. DADA2: High-Resolution Sample Inference from Illumina Amplicon Data. *Nature methods* **2016**, *13* (7), 581–583.
(7) Callahan, B. J.; Sankaran, K.; Fukuyama, J. A.; McMurdie, P. J.; Holmes, S. P. Bioconductor Workflow for Microbiome Data Analysis: From Raw Reads to Community Analyses. *F1000Research* **2016**, *5*.
(8) McLaren MR; Callahan BJ. Silva 138.1 Prokaryotic SSU Taxonomic Training Data Formatted for DADA2. https://doi.org/10.5281/zenodo.4587955.
(9) Quast, C.; Pruesse, E.; Yilmaz, P.; Gerken, J.; Schweer, T.; Yarza, P.; Peplies, J.; Glöckner, F. O. The SILVA Ribosomal RNA Gene Database Project: Improved Data Processing and Web-Based Tools. *Nucleic acids research* **2012**, *41* (D1), D590–D596.
(10) TreeSummarizedExperiment v2.8. https://doi.org/10.12688/f1000research.26669.2 (accessed 2024-05-31).
(11) Mia - Microbiome Analysis. https://github.com/microbiome/mia (accessed 2024-05-31).
(12) Vegan v2.6-4. https://CRAN.R-project.org/package=vegan (accessed 2024-05-31).
(13) Phyloseq v1.44. https://doi.org/10.1371/journal.pone.0061217 (accessed 2024-05-31).
(14) Microbiome v1.22. http://microbiome.github.io (accessed 2024-05-31).
(15) MicroViz v0.10.8. https://doi.org/10.21105/joss.03201 (accessed 2024-05-31).
(16) Fernandes, A. D.; Reid, J. N.; Macklaim, J. M.; McMurrough, T. A.; Edgell, D. R.; Gloor, G. B. Unifying the Analysis of High-Throughput Sequencing Datasets: Characterizing RNA-Seq, 16S RRNA Gene Sequencing and Selective Growth Experiments by Compositional Data Analysis. *Microbiome* **2014**, *2*, 1–13.
(17) Love, M. I.; Huber, W.; Anders, S. Moderated Estimation of Fold Change and Dispersion for RNA-Seq Data with DESeq2. *Genome biology* **2014**, *15*, 1–21.
